# Analysis of traditional knowledge of medicinal plants from residents in Gayasan National Park (Korea)

**DOI:** 10.1186/1746-4269-10-74

**Published:** 2014-10-21

**Authors:** Mi-Jang Song, Hyun Kim, Byoung-Yoon Lee, Heldenbrand Brian, Chan-Ho Park, Chang-Woo Hyun

**Affiliations:** Department of Integrated Bioresource Science, Graduate School of Jeonju University, 303 Cheonjam-ro, Wansan-gu, Jeonju, 560-759 Republic of Korea; School of Alternative Medicine and Health Science, Jeonju University, 303 Cheonjam-ro, Wansan-gu, Jeonju, 560-759 Republic of Korea; Department of Biological Resources, National Institute of Biological Resources, Incheon, 404-708 Republic of Korea; School of Liberal Arts, Jeonju University, 303 Cheonjam-ro, Wansan-gu, Jeonju, 560-759 Republic of Korea

**Keywords:** Traditional knowledge, Informant consensus factor, Fidelity level, Inter-network analysis, Gayasan National Park

## Abstract

**Background:**

The purpose of this study is to investigate and analyze the traditional knowledge of medicinal plants used by residents in Gayasan National Park in order to obtain basic data regarding the sustainable conservation of its natural plant ecosystem.

**Methods:**

Data was collected using participatory observations and in-depth interviews, as the informants also become investigators themselves through attending informal meetings, open and group discussions, and overt observations with semi-structured questionnaires. Quantitative analyses were accomplished through the informant consensus factor (ICF), fidelity level, and inter-network analysis (INA).

**Results:**

In total, 200 species of vascular plants belonging to 168 genera and 87 families were utilized traditionally in 1,682 ethnomedicianal practices. The representative families were Rosaceae (6.5%) followed by Asteraceae (5.5%), Poaceae (4.5%), and Fabaceae (4.0%). On the whole, 27 kinds of plant-parts were used and prepared in 51 various ways by the residents for medicinal purposes. The ICF values in the ailment categories were muscular-skeletal disorders (0.98), pains (0.97), respiratory system disorders (0.97), liver complaints (0.97), and cuts and wounds (0.96). In terms of fidelity levels, 57 plant species showed fidelities levels of 100%. Regarding the inter-network analysis (INA) between ailments and medicinal plants within all communities of this study, the position of ailments is distributed into four main groups.

**Conclusion:**

The results of the inter-network analysis will provide a suitable plan for sustainable preservation of the national park through a continued study of the data. Particular species of medicinal plants need to be protected for a balanced plant ecosystem within the park. Consequently, through further studies using these results, proper steps need to be established for preparing a wise alternative to create a sustainable natural plant ecosystem for Gayasan National Park and other national parks.

## Introduction

National Parks in the world grow various useful bio-resources owing to its well-preserved natural plant ecosystem. For residents living within a national park, these resources become materials for medicine, food, clothing, and other purposes. The relationship between the natural conservation of a national park and the life of its residents is interconnected [1].

Also, the traditional knowledge regarding the bio-resources of residents living in a national park affects the natural conservation of an ecosystem [2]. Among all knowledge, the traditional knowledge of food and medicinal plants causes negative effects to plant ecosystems because of its applicability. Particularly, the additional value of medicinal plants is a major concern for species possessing high efficacy and utility as they are potentially overused and supplies become rapidly depleted [3]. These trends are stronger in developing countries with poor health care systems than in developed countries [4].

From this point, the analysis and investigation of traditional knowledge for medicinal plants used by residents in a national park will be applied for a sustainable conservation plan for a natural plant ecosystem.

Research in this field has been widely accomplished in several countries around the world, including Europe [5, 6], Africa [7, 8], Asia [9, 10], North America [11], and South America [12]. The results of this research have been utilized as basic data to formulate a sustainable conservation plan for natural plant ecosystems within a national park.

In Korea, the research of the same pattern has been only conducted from residents living in Hallasan National Park [13].

The natural plant ecosystem of Gayasan National Park, located in the southeast region of Korea has been well preserved. Residents have maintained a traditional culture for over 30 years. These residents have utilized various ethnomedicine for treating numerous ailments. Therefore, a balance between medicinal plants and their utilization plays a very important role to sustain the natural conservation of a national park.

This study aims to investigate and analyze the traditional knowledge of medicinal plants used by residents in Gayasan National Park in order to obtain basic data regarding the sustainable conservation of its natural plant ecosystem.

## Research area and methods

### Climate and geography of Gayasan National Park

**Figure 1:**
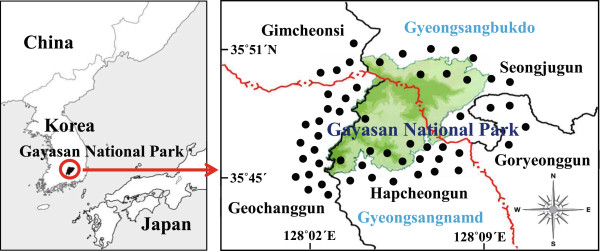
**Investigation sites.**

The study area is located in the center of the southern region of Korea and lies between 35° 45′N to 35° 51′N latitude and 128° 02′ 30″E to 128° 09′ 30″E longitude (Figure 1). The study area measures 77.256 km^2^ in areas including two provinces, one city, and four counties in its administrative district [14]. The annual average temperature is 13°C and the annual average precipitation is approximately 1,275.6 mm [15].

### Investigative method

Proper data was collected using participatory observations and in-depth interviews, as the informants also become investigators themselves through attending informal meetings, open and group discussions, and overt observations with semi-structured questionnaires [16, 17].

The content of the semi-structured questionnaires was composed of diverse information about medicinal plants, including local names, plant-parts used, ailments, methods of preparation, manufacturing and administration, dosage, and the usable duration regarding each curable formula [17–19].

All plant specimens were collected during their flowering or fruiting seasons and were organized utilizing the normal specimen manufacturing method [18, 20]. The voucher specimens were deposited for preservation in the herbarium of Jeonju University. The precise identification of plants mentioned by the informants was performed in accordance with Lee [21] and Lee [22]. Scientific names of plants were confirmed by the National Knowledge and Information System for Biological Species [23] of Korea.

### Quantitative analysis

#### Informant consensus factor (ICF)

The ICF was used to analyze the agreement degree of the informants’ knowledge about each category of ailments [24, 25]. The ICF was calculated using the following formula:


where *nur* stands for the number of use reports of informants for a particular illness category, and *nt* is the number of species used by all informants for a particular illness.

#### Fidelity level (FL)

The FL was employed to determine the most important plant species used for treating certain diseases by the local herbal practitioners and elderly people living in the study area [17, 20, 26]. The FL was calculated using the following formula:


where Np is the number of informants that mentioned the specific plant species used to treat certain ailments, and N is the total number of the informants who utilized the plants as medicine for treating any given ailment.

#### Inter-network analysis (INA)

The INA considers the results of the interrelationship among each individual of a community instead of focusing on the independent characteristics of an individual within the community. The INA has been applied within communities for various ethnographical problems, including ethnogenesis [27] and obesity [28–30].

However, the INA has yet to be applied to traditional medicinal knowledge, although it has been included in relation to its ethnographical properties.

Therefore, this research has newly applied this method in order to attain more network information between categories of ailments and medicinal plants within communities in Korea. The results of the INA were analyzed using UCINET (Ver. 6.460) and NetDraw (Ver. 2.125) software [31, 32].

## Results and discussion

### Demographic characteristics of the informants

Field investigations were conducted from May 2012 to October 2012. All 208 key informants (28 men and 180 women) were randomly selected at 54 sites, which included community halls, the senior welfare centers, and the traditional markets. The average age of the informants was 77 years of age, with a range in age from 52 to 93, with residents living more than 30 years in the study area (Table 1).

**Table 1:** **Demographic characteristics**

Gender	
Male	28 (13.5%)
Female	180 (86.5%)
Age	
50-59	1 (0.5%)
60-69	21 (10.1%)
70-79	106 (51.0%)
80-89	74 (35.6%)
90-99	6 (2.9%)
Educational attainment	
Never attended school	154 (74.0%)
Attended school for less than 6 years	13 (6.3%)
Attended school for 6 years	17 (8.2%)
Finished middle school	14 (6.7%)
Finished high school	10 (4.8%)

### Ethnomedicinal knowledge

In total, 200 species of vascular plants belonging to 168 genera and 87 families were utilized traditionally in 1,682 ethnomedicianal practices.

The species numbers from the Gayasan National Park were similar to those of other communities from previous research, including the communities of the western region of Jeolla North Province (183 species), the southern mountainous region (217 species) of Korea, and Hallasan National Park [13, 16, 19]. These results conclude that people living in the communities in and around Gayasan National Park possess similar application abilities for ethnomedicinal knowledge with other communities in Korea. Also, the number of species recorded in Gayasan National Park occupied 36.9% of the flora (542 species) in the study area [15]. This high percentage means that all medicinal plants collected by residents may negatively affect the conservation of a natural plant ecosystem.

Regarding the number of species in their families and the percentage of the number of times mentioned from the our current investigations, 13 species of Rosaceae occupied 6.5% of the total species mentioned, followed by 12 species of Asteraceae, 11 species of Poaceae, and 9 species of Fabaceae, which occupied 5.5%, 4.5% and 4.0% of the whole, respectively. Generally, these four families contain many more medicinal species than any other family. This data is similar to results obtained within national parks of other countries, including Pollino National Park in Italy [5], Kibale National Park in Uganda [8], Ben En National Park in Vietnam [9], Cumbres de Monterrey National Park in Mexico [11], and Isiboro-Secure National Park in Bolivia [12].

Our overall analysis reveals that 27 plant-parts were selected as medicinal materials. Stems were the most frequently used plant-parts, constituting 25.7% of the whole followed by roots (22.7%), fruits (12.9%), whole parts (7.3%), seeds (5.3%), and other sections of the plant. These results were similar to data obtained in Korea for the western region of Jeolla North Province [19], the southern mountainous regions [16], and Hallasan National Park [13].

The results revealed 51 modes of preparation for the medicinal materials. The percentages for the main modes of preparation were as follows: infusions (32.1%), sweet drinks made from fermented rice (20.1%), brewings (9.3%), macerations (7.1%), and juices (6.8%). Oral administration accounted for 84.8% of all applications, while topical application totaled 15.2%.

Among the medicinal plants mentioned more than 50 times, with certain species used to treat numerous ailments, *Hordeum vulgare* var. *hexastichon* (L.) Asch. was used to treat 36 ailments, followed by *Artemisia princeps* Pamp. in treating 24 ailments, and *Zanthoxylum schinifolium* Siebold. & Zucc. for treating 11 ailments. One area of major concern is the over application of the *Artemisia princeps* Pamp. and *Zanthoxylum schinifolium* Siebold. & Zucc. which can easily lead to its extinction within the plant ecosystem.

### Ethno-veterinary knowledge

The medicinal plants for ethno-veterinary treatments were recorded as 15 families, 22 genera, and 23 species that displayed 38 ethnomedicinal practices (Table 2). Among the veterinary medicinal plants, the whole part of *Artemisia princeps* Pamp., *Papaver somniferum* L., *Picrasma quassioides* (D.Don) Benn., *Sanguisorba officinalis* L., and *Poria cocos* Wolf were used most often to treat bovine diarrhea. Particularly, *Ipomoea batatas* (L.) Lam., *Ricinus communis* L., and *Zanthoxylum schinifolium* Siebold. & Zucc. were ingested for particular pig ailments, while *Lageneria leucantha* Rushy was generally used for treating a bovine disease caused by a loss of appetite.

**Table 2:** **Plant species for medicinal purposes in community of Gayasan National Park in Korea**

Family	Scientific name	Voucher	Abbreviation ^*^	Korean name	Used part	Ailments	Preparation	Application	FL
Aceraceae	*Acer pictum* subsp. *mono* (Maxim.) Ohashi	KH5340	S1	Gorosoenamu	Sap	Hypofunction	Sap	Oral	100.00
Actinidiaceae	*Actinidia arguta* (Siebold & Zucc.) Planch. ex Miq.	KH5341	S5	Darae	Stem	Abdominal pain	Infusion	Oral	100.00
Amaranthaceae	*Achyranthes japonica* (Miq.) Nakai	KH5342	S2	Soemureup	Root	Arm pain	A sweet drink made from fermented rice, brewing, infusion	Oral	16.71
						Arthritis	Infusion	Oral	1.62
						Bone diseases	A sweet drink made from fermented rice, brewing, infusion	Oral	32.61
						Common cold	A sweet drink made from fermented rice, infusion	Oral	2.43
						Convulsion	A sweet drink made from fermented rice, brewing, infusion	Oral	3.23
						Knee pain	A sweet drink made from fermented rice	Oral	2.16
						Leg pain	A sweet drink made from fermented rice, brewing, decoction, infusion	Oral	31.54
						Lumbago	A sweet drink made from fermented rice, infusion	Oral	9.16
						Pollakiuria	Infusion	Oral	0.54
	*Celosia cristata* L.	KH5343	S41	Maendeurami	Whole part	Carpal tunnel syndrome	Infusion	Oral	50.00
						Tarsal tunnel syndrome	Infusion	Oral	50.00
Anacardiaceae	*Rhus javanica* L.	KH5344	S155	Bulknamu	Worm cyst	Coated tongue	Infusion	Topical	15.12
						Stomatitis	Dried, infusion, maceration, powder, raw, steamed	Oral, topical	84.88
	*Rhus verniciflua* Stokes	KH5345	S156	Otnamu	Bark	Abdominal cold hypersentivity	Infusion, simmer	Oral	58.97
						Gastroenteric disorder	Simmer	Oral	19.87
					Lacquer	Abdominal cold hypersentivity	Mixed in egg, roast, sap	Oral	58.97
						Facial nerve paralysis	Raw	Topical	0.64
					Stem	Abdominal cold hypersentivity	Infusion, simmer, soup	Oral	58.97
						Diarrhea	Burn, powder	Oral	1.28
						Gastroenteric disorder	Infusion, simmer	Oral	19.87
						Hangover	Infusion, simmer	Oral	10.90
						Indigestion	Infusion	Oral	3.85
						Lack of energy	Infusion	Oral	1.92
						Woman diseases	Infusion	Oral	2.56
					Young leaf	Gastroenteric disorder	Raw	Oral	19.87
Apiaceae	*Angelica acutiloba* (Siebold & Zucc.) Kitag.	KH5346	S14	Waedanggwi	Root	Poor circulation	Infusion	Oral	53.33
						Lack of energy	Boiling, decoction	Oral	46.67
	*Angelica dahurica* (Fisch. ex Hoffm.) Benth. & Hook.f. ex Franch. & Sav.	KH5347	S15	Guritdae	Root	Lack of energy	Decoction	Oral	100.00
	*Angelica gigas* Nakai	KH5348	S16	Chamdanggwi	Root	Lack of energy	Decoction, infusion	Oral, topical	100.00
	*Bupleurum falcatum* L.	KH5349	S32	Siho	Root	Alopecia	A sweet drink made from fermented rice	Oral	100.00
	*Cnidium officinale* Makino	KH5350	S48	Cheongung	Root	Bone diseases	A sweet drink made from fermented rice	Oral	23.53
						Lack of energy	Boiling, decoction	Oral, topical	52.94
						Woman diseases	Decoction	Oral	23.53
	*Ledebouriella seseloides* (Hoffm.) H.Wolff	KH5351	S99	Bangpung	Root	Hypofunction	Decoction	Oral	100.00
	*Oenanthe javanica* (Blume) DC.	KH5352	S111	Minari	Stem	Cancer	Juice	Oral	25.00
						Liver diseases	Juice	Oral	29.17
						Pus	Maceration	Topical	25.00
						Snakebite	Maceration	Topical	20.83
					Whole part	Liver diseases	Juice	Oral	29.17
	*Ostericum praeteritum* Kitag.	KH5353	S115	Ganghwal	Root	Hyperthermia	Infusion	Oral	60.00
						Lack of energy	Decoction	Oral	40.00
Araceae	*Arisaema amurense* f. *serratum* (Nakai) Kitag.	KH5354	S19	Cheonnamseong	Corm	Chronic myofascial pain	Chopped noodles, clear soup with flour dumplings	Oral	55.56
						Extravasated blood	Maceration	Topical	3.70
						Milk fever	Clear soup with flour dumplings	Oral	12.96
						Tonsillitis	Clear soup with flour dumplings	Oral	14.81
						Wound	Maceration	Topical	12.96
					Stem	Tonsillitis	Infusion	Oral	14.81
	*Colocasia esculenta* (L.) Schott	KH5355	S52	Toran	Flower	Sterility	Infusion	Oral	100.00
Araliaceae	*Aralia cordata* var. *continentalis* (Kitag.) Y.C.Chu	KH5356	S17	Dokhwal	Root	Abdominal pain	Infusion	Oral	5.56
						Anorexia	Infusion	Oral	5.56
						Arthritis	Infusion	Oral	11.11
						Bone diseases	Brewing	Oral	14.81
						Chronic myofascial pain	Brewing	Oral	18.52
						Gastroenteric disorder	Dissolution, maceration	Oral	11.11
						Lumbago	Brewing, maceration	Oral	33.33
	*Aralia elata* (Miq.) Seem.	KH5357	S18	Dureupnamu	Root	Edema	Infusion	Oral	100.00
	*Eleutherococcus senticosus* (Rupr. & Maxim.) Maxim.	KH5358	S66	Gasiogalpi	Stem	Bone diseases	Decoction	Oral	100.00
	*Eleutherococcus sessiliflorus* (Rupr. & Maxim.) S.Y.Hu	KH5359	S67	Ogalpinamu	Fruit	Bone diseases	Extraction	Oral	47.38
						Arm pain	A sweet drink made from fermented rice, brewing, infusion	Oral	11.53
						Arthritis	A sweet drink made from fermented rice	Oral	1.05
						Bone diseases	A sweet drink made from fermented rice, brewing, infusion	Oral	47.38
						Glycosuria	Infusion	Oral	1.05
						Growing pain	A sweet drink made from fermented rice, brewing	Oral	2.94
						Hyperthermia	Brewing	Oral	0.21
						Leg pain	A sweet drink made from fermented rice, brewing, infusion	Oral	22.01
						Liver diseases	Infusion	Oral	1.89
						Lumbago	A sweet drink made from fermented rice, infusion	Oral	9.64
						Neuralgia	Infusion	Oral	0.42
						Paralysis	Infusion	Oral	1.26
						Shoulder pain	A sweet drink made from fermented rice	Oral	0.42
						Sinews and joint pain	A sweet drink made from fermented rice	Oral	0.21
	*Kalopanax septemlobus* (Thunb.) Koidz.	KH5360		Eumnamu		Arm pain	A sweet drink made from fermented rice	Oral	9.76
						Bone diseases	Brewing, infusion	Oral	55.10
						Leg pain	A sweet drink made from fermented rice	Oral	17.57
					Stem	Abdominal cold hypersentivity	Infusion, simmer	Oral	0.65
						Arm pain	A sweet drink made from fermented rice, brewing, Infusion, Simmer	Oral	9.76
						Bone diseases	A sweet drink made from fermented rice, brewing, infusion, simmer	Oral	55.10
						Gastroenteric disorder	A sweet drink made from fermented rice, brewing	Oral	3.04
						Hyperthermia	Brewing	Oral	0.22
						Indigestion	Infusion	Oral	0.22
						Lack of energy	Infusion	Oral	0.87
						Leg pain	A sweet drink made from fermented rice, brewing, infusion, simmer	Oral	17.57
						Liver diseases	Infusion	Oral	0.87
						Lumbago	A sweet drink made from fermented rice, infusion	Oral	9.11
						Neuralgia	Infusion	Oral	0.43
						Paralysis	Brewing, infusion	Oral	2.17
Aristolochiaceae	*Asarum sieboldii* Miq.	KH5361	S24	Jokdoripul	Root	Dental pain	Chew, dried	Topical	22.22
						Pain	A sweet drink made from fermented rice, infusion	Oral	77.78
Aspleniaceae	*Athyrium yokoscense* (Franch. & Sav.) H.Christ	KH5362	S26	Baemgosari	Stem	Abrasion	Maceration, rubbing	Topical	13.41
						Common cold	Infusion	Oral	6.10
						Hemorrhaging	Maceration, rubbing	Topical	62.20
						Snakebite	Maceration, raw	Topical	15.85
						Wound	Maceration	Topical	2.44
Asteraceae	*Artemisia capillaris* Thunb.	KH5363	S21	Sacheolssuk	Aerial part	Abdominal cold hypersentivity	Infusion	Oral	2.18
						Boils	Decoction	Oral	0.87
						Cattle disease	Burn, dried, fumigation	Topical	0.87
						Glycosuria	Infusion	Oral	1.75
						Jaundice	A sweet drink made from fermented rice, grain syrup, infusion, pill, simmer	Oral	67.25
						Liver diseases	A sweet drink made from fermented rice, decoction, grain syrup, infusion, juice, simmer	Oral	20.96
					Leaf	Indigestion	Decoction	Oral	0.87
						Jaundice	A sweet drink made from fermented rice, decoction, dried, infusion	Oral	67.25
						Liver diseases	Infusion	Oral	20.96
						Pollakiuria	Infusion	Oral	1.75
					Stem	Indigestion	Decoction	Oral	0.87
						Jaundice	Decoction	Oral	67.25
					Whole part	Gastroenteric disorder	A sweet drink made from fermented rice, infusion, pill	Oral	3.49
						Jaundice	A sweet drink made from fermented rice, infusion	Oral	67.25
						Liver diseases	A sweet drink made from fermented rice, pill	Oral	20.96
	*Artemisia montana* (Nakai) Pamp.	KH5364	S22	Sanssuk	Leaf	Leg pain	Rubbing	Topical	50.00
						Lumbago	Rubbing	Topical	50.00
	*Artemisia princeps* Pamp.	KH5365	S23	Ssuk	Aerial part	Abdominal pain	Juice	Oral	16.35
						Bone diseases	A sweet drink made from fermented rice	Oral	1.26
						Cattle disease	Burn, dried, fumigation	Topical	1.26
						Diarrhea	Infusion, juice	Oral	6.29
						Dysentery	Infusion, juice	Oral	2.52
						Head laceration	Raw	Topical	1.26
						Hemorrhage	Maceration, rubbing	Topical	38.36
						Hookworm	Raw	Topical	0.63
						Irregular menstruation	Decoction, infusion, pill	Oral	0.63
					Leaf	Abdominal pain	Juice, maceration	Oral	16.35
						Anorexia	Juice	Oral	2.83
						Arthritis	Dried, powder	Topical	1.26
						Burn	Maceration	Topical	0.31
						Diarrhea	Juice, raw	Oral	6.29
						Epistaxis	Maceration, rubbing	Topical	4.09
						Gastroenteric disorder	Powder	Topical	5.66
						Hemorrhaging	Maceration, powder, rubbing	Topical	38.36
						Hoove	Raw	Oral	2.20
						Indigestion	Juice, raw	Oral	2.52
						Jaundice	Rubbing	Topical	1.57
						Pus	Powder	Topical	1.26
						Overheating	Juice	Oral	0.94
						Wound	Rubbing	Topical	6.60
					Root	Woman diseases	Infusion	Oral	2.20
					Whole part	Abdominal pain	Juice	Oral	16.35
						Anorexia	Juice	Oral	2.83
						Diarrhea	Juice	Oral	6.29
						Dysentery	Juice	Oral	2.52
						Gastroenteric disorder	Infusion, juice	Oral	5.66
						Hemorrhage	Maceration, rubbing	Topical	38.36
						Indigestion	Juice	Oral	2.52
	*Atractylodes ovata* (Thunb.) DC.	KH5366	S28	Sapju	Root	Arm pain	Decoction	Oral	1.09
						Bone diseases	Decoction	Oral	1.09
						Gastroenteric disorder	A sweet drink made from fermented rice, decoction, dried, infusion maceration, pill, powder, raw, steep in rice water	Oral	52.17
						Indigestion	Dried, maceration, powder, raw, steep in rice water	Oral	6.52
						Lack of energy	A sweet drink made from fermented rice, boiling, decoction, dried, infusion, pill, powder, steep in rice water	Oral	38.04
						Leg pain	Decoction	Oral	1.09
	*Carthamus tinctorius* L.	KH5367	S37	Itggot	Seed	Bone diseases	A sweet drink made from fermented rice	Oral	33.33
						Fracture	Boiling	Oral	33.33
						Lumbago	A sweet drink made from fermented rice	Oral	33.33
	*Cirsium japonicum* var. *maackii* (Maxim.) Matsum.	KH5368	S45	Eonggeongkwi	Root	Abdominal cold hypersentivity	Juice	Oral	1.80
						Abdominal pain	Juice	Oral	0.90
						Anhidrosis	Infusion, juice	Oral	4.50
						Bone diseases	Juice	Oral	0.90
						Epistaxis	Juice	Oral	0.90
						Gastroenteric disorder	Juice	Oral	0.90
						Hangover	Juice	Oral	3.15
						Hookworm	Juice	Oral	2.70
						Lack of energy	Juice	Oral	3.60
						Lumbago	Infusion, juice	Oral	4.50
						Sexual enhancement	Infusion, juice	Oral	75.23
						Sterility	Infusion	Oral	0.90
	*Helianthus tuberosus* L.	KH5369	S86	Ddungddanji	Tuber	Glycosuria	Maceration, tea	Oral	100.00
	*Lactuca sativa* L.	KH5370	S97	Sangchu	Seed	Oligogalactia	Infusion	Oral	100.00
	*Petasites japonicus* (Siebold & Zucc.) Maxim.	KH5371	S123	Meowi	Leaf	Gastroenteric disorder	Infusion, wrapped in leaves	Oral	27.59
						Lacquer poisoning	Maceration	Topical	37.93
						Paralysis	Maceration	Oral	10.34
					Root	Convulsion	Maceration	Oral	6.90
						Leg pain	Brewing	Oral	17.24
					Stem	Gastroenteric disorder	Seasoned cooked vegetables	Oral	27.59
	*Sigesbeckia glabrescens* (Makino) Makino	KH5372	S175	Jindeukchal	Aerial part	Pruritus	A sweet drink made from fermented rice, brewing, infusion, pill, simmer	Oral, topical	79.89
					Flower	Hypertension	Grain syrup, infusion	Oral	3.45
						Postpartum myofascial pain syndrome	A sweet drink made from fermented rice, infusion	Oral	11.49
						Pruritus	Dried, mixed in liquor, maceration, pill	Oral	79.89
					Fruit	Pruritus	Brewing, infusion	Oral, topical	79.89
					Leaf	Postpartum myofascial pain syndrome	A sweet drink made from fermented rice, infusion	Oral	11.49
					Whole part	Bone diseases	A sweet drink made from fermented rice	Oral	5.17
						Pruritus	A sweet drink made from fermented rice, infusion	Oral, topical	79.89
	*Taraxacum platycarpum* Dahlst.	KH5373	S184	Mindeulre	Leaf	Liver diseases	Dried, infusion, juice, Kimchi, raw, seasoned cooked vegetables	Oral	91.76
					Root	Cancer	Infusion	Oral	8.24
					Whole part	Cancer	Dried, infusion	Oral	8.24
						Liver diseases	A sweet drink made from fermented rice, dried, extraction, infusion, juice	Oral	91.76
	*Xanthium strumarium* L.	KH5374	S195	Doggomari	Aerial part	Boils	Infusion	Topical	22.22
					Fruit	Pruritus	Infusion	Topical	40.74
					Stem	Boils	Infusion	Topical	22.22
					Whole part	Boils	Infusion	Topical	22.22
						Gastroenteric disorder	A sweet drink made from fermented rice	Oral	11.11
						Leg pain	A sweet drink made from fermented rice	Oral	11.11
						Pruritus	A sweet drink made from fermented rice, brewing, infusion	Oral, topical	40.74
						Skin diseases	A sweet drink made from fermented rice, brewing, infusion	Oral, topical	14.81
Balsaminaceae	*Impatiens balsamina* L.	KH5375	S91	Bongseonhwa	Whole part	Indigestion	Infusion	Oral	59.26
						Sterility	Decoction, infusion	Oral	40.74
Berberidaceae	*Epimedium koreanum* Nakai	KH5376	S68	Samjigueopcho	Aerial part	Sexual enhancement	A sweet drink made from fermented rice, infusion	Oral	57.14
						Sterility	Tea	Oral	42.86
Betulaceae	*Betula costata* Trautv.	KH5377	S29	Geojesunamu	Sap	Hypofunction	Sap	Oral	35.71
						Indigestion	Sap	Oral	7.14
					Stem	Arm pain	A sweet drink made from fermented rice	Oral	28.57
						Leg pain	A sweet drink made from fermented rice	Oral	28.57
Bignoniaceae	*Catalpa ovata* G.Don	KH5378	S39	Gaeodong	Stem	Bone diseases	A sweet drink made from fermented rice	Oral	30.77
						Indigestion	Infusion	Oral	7.69
						Leg pain	A sweet drink made from fermented rice	Oral	30.77
						Lumbago	A sweet drink made from fermented rice	Oral	30.77
Boraginaceae	*Lithospermum erythrorhizon* Siebold & Zucc.	KH5379	S102	Jichi	Root	Bruising	Infusion, poultice	Oral, topical	10.00
						Convulsion	Decoction, infusion, oil, panbroiled, poultice, roast	Oral, topical	83.33
						Indigestion	Panbroiled	Oral	6.67
Brassicaceae	*Brassica rapa* var. *glabra* Regel	KH5380	S30	Baechu	Leaf	Burn	Kimchi, raw	Topical	100.00
	*Capsella bursapastoris* (L.) L.W.Medicus	KH5381	S34	Naengi	Whole part	Liver diseases	Juice, seasoned cooked vegetables, soup	Oral	100.00
	*Raphanus sativus* L.	KH5382	S151	Mu	Leaf	Lumbago	Steamed	Topical	1.72
					Tuberous root	Bronchitis	Infusion	Oral	4.31
						Common cold	Decoction, extraction, fermentation, infusion, raw, roast	Oral	56.90
						Cough	Infusion, raw, roast	Oral	37.07
Campanulaceae	*Adenophora triphylla* var. *japonica* (Regel) H. Hara	KH5383	S6	Jandae	Root	Sterility	Infusion	Oral	20.00
						Woman diseases	A sweet drink made from fermented rice, raw, simmer	Oral	80.00
	*Codonopsis lanceolata* (Siebold & Zucc.) Trautv.	KH5384	S50	Deodeok	Root	Abdominal cold hypersentivity	Maceration, raw	Oral	17.39
						Bronchitis	Maceration, raw	Oral	19.57
						Cough	Steamed	Oral	10.87
						Orchiopathy	Brewing, maceration, raw	Oral	26.09
						Sexual enhancement	Brewing, infusion	Oral	26.09
	*Codonopsis ussuriensis* (Rupr. & Maxim.) Hemsl.	KH5385	S51	Sogyeongbulal	Root	Sexual enhancement	Infusion	Oral	100.00
	*Platycodon grandiflorum* (Jacq.) A.DC.	KH5386	S134	Doraji	Flower	Sterility	Infusion	Oral	2.62
					Root	Abdominal pain	Infusion	Oral	1.12
						Anorexia	Infusion	Oral	1.12
						Asthma	Brewing	Oral	11.99
							Dried, fermentation, infusion, powder, pill	Oral	
						Bronchitis	Extraction, infusion, juice, powder	Oral	7.12
						Common cold	Brewing, decoction, dried, infusion, maceration, mixed in honey, pill, powder	Oral	21.72
						Cough	Brewing, decoction, dissolution, dried, extraction, fermentation, infusion, maceration, mixed in honey, powder, pill	Oral	50.56
						Hyperthermia	Infusion	Oral	1.50
						Leukorrhea	Infusion	Oral	1.50
						Sinews and joint pain	Infusion	Oral	0.75
						Sterility	Infusion	Oral	2.62
Cannabaceae	*Cannabis sativa* L.	KH5387	S33	Sam	Leaf	Paralysis	Infusion	Oral	100.00
	*Humulus japonicus* Sieboid & Zucc.	KH5388	S90	Hwansamdeonggul	Aerial part	Athlete’s foot	Juice	Topical	100.00
Caprifoliaceae	*Lonicera japonica* Thunb.	KH5389	S103	Indongdeonggul	Aerial part	Bone diseases	A sweet drink made from fermented rice	Oral	36.11
					Flower	Lack of energy	Brewing	Oral	1.39
					Stem	Arm pain	A sweet drink made from fermented rice, brewing, decoction, infusion	Oral	19.79
						Bone diseases	A sweet drink made from fermented rice, brewing, decoction, infusion	Oral	36.11
						Bronchitis	Infusion	Oral	0.35
						Carpal tunnel syndrome	Infusion	Oral	1.04
						Lack of energy	A sweet drink made from fermented rice	Oral	1.39
						Leg pain	A sweet drink made from fermented rice,, brewing, decoction, infusion	Oral	26.04
						Lumbago	A sweet drink made from fermented rice, infusion	Oral	14.24
						Tarsal tunnel syndrome	Infusion	Oral	1.04
Caryophyllaceae	*Dianthus longicalyx* Miq.	KH5390	S60	Sulpaeraengikkot	Whole part	Pollakiuria	Infusion	Oral	100.00
Celastraceae	*Euonymus alatus* (Thunb.) Siebold	KH5391	S70	Hwasalnamu	Leaf	Gastroenteric disorder	Seasoned cooked vegetables	Oral	100.00
					Stem	Gastroenteric disorder	Decoction	Oral	100.00
	*Euonymus sachalinensis* (F.Schmidt) Maxim.	KH5392	S71	Hoenamu	Leaf	Hemorrhoids	Infusion	Topical	100.00
					Stem	Hemorrhoids	Fumigation, infusion	Topical	100.00
Chenopodiaceae	*Kochia scoparia* (L.) Schrad. var. *scoparia*	KH5393	S96	Daepssari	Aerial part	Pollakiuria	Infusion	Oral	100.00
Commelinaceae	*Commelina communis* L.	KH5394	S53	Dalkuijangpul	Whole part	Bruising	Maceration	Topical	33.33
						Snakebite	Maceration	Topical	33.33
						Sprain	Maceration	Topical	33.33
Convolvulaceae	*Ipomoea batatas* (L.) Lam.	KH5395	S93	Goguma	Root	Pig disease	Raw	Oral	100.00
Cornaceae	*Cornus officinalis* Siebold & Zucc.	KH5396	S55	Sansuyu	Fruit	Lack of energy	Decoction	Oral	53.85
						Pollakiuria	Infusion	Oral	46.15
Crassulaceae	*Orostachys japonica* (Maxim.) A.Berger	KH5397	S112	Bawisol	Aerial part	Cancer	Maceration	Oral	75.00
					Whole part	Cancer	Dried, infusion	Oral	75.00
						Snakebite	Maceration	Topical	25.00
	*Sedum sarmentosum* Bunge	KH5398	S171	Dolnamul	Whole part	Gastroenteric disorder	Watery plain Kimchi	Oral	13.21
						Hepatitis	Juice	Oral	16.98
						Liver diseases	Maceration, powder, raw, watery plain Kimchi	Oral	52.83
						Snakebite	Maceration, raw	Topical	16.98
Cucurbitaceae	*Citrullus vulgaris* Schrad.	KH5399	S46	Subak	Fruit	Overheating	Extraction	Oral	100.00
	*Cucumis melo* var. *makuwa* Makino	KH5400	S56	Chamoe	Peduncle	Jaundice	Powder	Topical	55.56
						Otalgia	Powder	Topical	44.44
	*Cucumis sativus* L.	KH5401	S57	Oi	Stem	Pollakiuria	Dried, infusion	Oral	100.00
	*Cucurbita moschata* Duchesne	KH5402	S58	Hobak	Fruit	Asthma	Simmer	Oral	11.11
						Edema	Infusion, roast	Oral	61.11
						Gastroenteric disorder	Infusion	Oral	27.78
	*Lagenaria leucantha* Rusby	KH5403	S98	Bak	Endodermis	Cattle disease	Infusion	Oral	56.25
					Fruit	Cattle disease	Infusion	Oral	56.25
						Common cold	Infusion	Oral	31.25
					Pericarp	Pollakiuria	Infusion	Oral	12.50
	*Luffa cylindrica* Roem.	KH5404	S104	Susemioi	Fruit	Asthma	Extraction, sap	Oral	19.51
						Bronchitis	Extraction	Oral	10.98
						Chronic cough	Extraction, sap	Oral	4.88
						Cough	A sweet drink made from fermented rice, extraction, fermentation, infusion, oil, pan fried, sap	Oral	43.90
						Skin diseases	Extraction, sap	Oral, Topical	15.85
					Stem	Asthma	Sap	Oral	19.51
						Atopic dermatitis	Raw	Topical	4.88
						Bronchitis	Sap	Oral	10.98
						Skin diseases	Raw	Topical	15.85
	*Trichosanthes kirilowii* Maxim.	KH5405	S186	Haneultari	Fruit	Bruising	Dough	Topical	4.69
						Carpal tunnel syndrome	Dough	Topical	1.56
						Chronic myofascial pain	Brewing, infusion	Oral	37.50
						Lumbago	Brewing, infusion	Oral	12.50
						Neuralgia	Infusion	Oral	9.38
						Overheating	Extraction, infusion	Oral	18.75
						Tarsal tunnel syndrome	Dough	Topical	1.56
						Urticaria	Infusion	Oral	9.38
					Root	Induced abortion	Maceration	Oral	4.69
						Neuralgia	Infusion	Oral	9.38
						Overheating	Infusion	Oral	18.75
					Stem	Neuralgia	Infusion	Oral	9.38
Dioscoreaceae	*Dioscorea batatas* Decne.	KH5406	S61	Ma	Root	Gastroenteric disorder	Maceration, oil, raw	Oral	37.14
						Lack of energy	Maceration, oil, raw	Oral	34.29
						Milk fever	Maceration	Oral, topical	17.14
						Osteoporosis	Maceration	Oral	11.43
	*Dioscorea japonica* Thunb.	KH5407	S62	Chamma	Root	Gastroenteric disorder	Raw	Oral	100.00
Ebenaceae	*Diospyros kaki* Thunb.	KH5408	S63	Gamnamu	Fruit	Boils	Raw	Topical	4.05
						Cough	Ripe persimmon	Oral	6.76
						Diarrhea	Raw, roast	Oral	9.46
						Indigestion	Infusion, raw	Oral	47.30
					Peduncle	Acute gastroenteritis	Infusion	Oral	6.76
						Hiccups	Decoction, infusion	Oral	18.92
						Indigestion	Infusion	Oral	47.30
					Pericarp	Common cold	Infusion	Oral	6.76
Elaeagnaceae	*Elaeagnus umbellata* Thunb.	KH5409	S65	Borisunamu	Fruit	Asthma	Raw	Oral	11.59
						Bronchitis	Extraction	Oral	4.35
						Common cold	Extraction	Oral	5.80
						Cough	A sweet drink made from fermented rice, brewing, extraction, raw	Oral	75.36
					Stem	Asthma	A sweet drink made from fermented rice	Oral	11.59
						Cough	A sweet drink made from fermented rice, infusion	Oral	75.36
						Lumbago	A sweet drink made from fermented rice	Oral	2.90
Ericaceae	*Rhododendron mucronulatum* Turcz.	KH5410	S153	Jindalrae	Stem	Bone diseases	A sweet drink made from fermented rice	Oral	100.00
	*Rhododendron weyrichii* Maxim.	KH5411	S154	Chamkkoknamu	Root	Cough	Extraction	Oral	100.00
Eucommiaceae	*Eucommia ulmoides* Oliv.	KH5412	S69	Duchung	Bark	Arm pain	A sweet drink made from fermented rice	Oral	21.05
						Bone diseases	A sweet drink made from fermented rice	Oral	21.05
						Cancer	Infusion	Oral	10.53
						Leg pain	A sweet drink made from fermented rice	Oral	21.05
						Lumbago	A sweet drink made from fermented rice, decoction	Oral	26.32
Euphorbiaceae	*Ricinus communis* L.	KH5413	S157	Pimaja	Aerial part	Lumbago	A sweet drink made from fermented rice	Oral	2.80
					Fruit	Constipation	Oil, pan fried	Oral, topical	42.66
						Cough	Oil, pan fried	Oral	2.80
						Dental pain	Roast	Topical	3.50
						Indigestion	Oil, pan fried	Oral	44.06
						Pig disease	Oil	Oral	1.40
					Leaf	Otalgia	Infusion	Topical	2.80
Fabaceae	*Albizia julibrissin* Durazz.	KH5414	S8	Jagwinamu	Stem	Arm pain	A sweet drink made from fermented rice	Oral	18.18
						Bone diseases	A sweet drink made from fermented rice, brewing	Oral	54.55
						Leg pain	A sweet drink made from fermented rice	Oral	18.18
						Neuralgia	Infusion	Oral	9.09
	*Astragalus mongholicus* Bunge	KH5415	S27	Hwanggi	Root	Afterpain	Decoction, infusion, simmer	Oral	25.00
						Bone diseases	A sweet drink made from fermented rice	Oral	15.63
						Hyperridrosis	Boiling, infusion	Oral	37.50
						Lumbago	Simmer	Oral	15.63
						Woman diseases	Simmer	Oral	6.25
	*Caragana sinica* (Buc'hoz) Rehder	KH5416	S36	Goldamcho	Leaf	Chronic myofascial pain	Brewing, maceration	Oral, topical	9.00
						Leg pain	Brewing, maceration	Oral, topical	18.51
						Arm pain	A sweet drink made from fermented rice, brewing, infusion	Oral	12.85
						Arthritis	Infusion	Oral	1.54
						Bone diseases	A sweet drink made from fermented rice, brewing, infusion, juice	Oral	47.56
						Chronic myofascial pain	Brewing, juice, maceration	Oral, topical	9.00
						Gastroenteric disorder	A sweet drink made from fermented rice	Oral	1.03
						Leg pain	A sweet drink made from fermented rice, brewing, infusion, maceration	Oral, topical	18.51
						Lumbago	A sweet drink made from fermented rice, infusion, juice	Oral	8.48
						Neuralgia	A sweet drink made from fermented rice, brewing, infusion	Oral	1.03
	*Gleditsia sinensis* Lamarck	KH5417	S83	Jogakjanamu	Stem	Arm pain	A sweet drink made from fermented rice	Oral	26.92
						Bone diseases	A sweet drink made from fermented rice	Oral	11.54
						Leg pain	A sweet drink made from fermented rice	Oral	26.92
						Lumbago	A sweet drink made from fermented rice, infusion	Oral	19.23
						Shoulder pain	A sweet drink made from fermented rice	Oral	15.38
	*Glycine max* (L.) Merr.	KH5418	S84	Kong	Seed	Acute gastroenteritis	Raw	Oral	3.33
						Asthma	Oil	Oral	1.43
						Bee sting	Fermentation	Topical	0.95
						Carpal tunnel syndrome	A sweet drink made from fermented rice, infusion	Oral	3.81
						Common cold	Fermentation, roast	Oral	23.33
						Cough	Fermentation, infusion, oil, panbroiled, roast	Oral	19.05
						Hangover	Soup	Oral	0.48
						Head laceration	Fermentation	Topical	17.14
						Hemorrhaging	Boiling, fermentation, fumigation	Topical	5.24
						Milk fever	Infusion	Oral	15.24
						Snakebite	Fermentation	Topical	0.95
						Tarsal tunnel syndrome	A sweet drink made from fermented rice, infusion	Oral	3.81
						Urticaria	Infusion	Oral	2.86
						Whitlow	Boiling	Topical	1.90
						Wound	Fermentation	Topical	0.48
					Young leaf	Common cold	A sweet drink made from fermented rice, extraction, fermentation, infusion, roast	Oral	23.33
						Milk fever	Fumigation, infusion, soup	Oral, topical	15.24
	*Glycyrrhiza uralensis* Fisch.	KH5419	S85	Gamcho	Root	Abdominal cold hypersentivity	Infusion	Oral	19.23
						Abdominal pain	Infusion	Oral	5.77
						Anorexia	Infusion	Oral	5.77
						Bone diseases	A sweet drink made from fermented rice, infusion	Oral	38.46
						Common cold	Decoction	Oral	11.54
						Gastroenteric disorder	Decoction	Oral	1.92
						Osteoporosis	Infusion	Oral	5.77
						Poisonings	Infusion	Oral	5.77
						Sterility	Infusion	Oral	5.77
	*Pueraria lobata* (Willd.) Ohwi	KH5420	S143	Chilk	Flower	Abdominal pain	Decoction	Oral	15.89
						Hangover	Decoction	Oral	49.53
					Root	Abdominal pain	Grain syrup, infusion	Oral	15.89
						Anorexia	Infusion	Oral	2.80
						Gastroenteric disorder	Boiled rice, brewing, decoction, infusion, juice	Oral	14.95
						Hangover	Clear soup with flour dumplings, grain syrup, infusion, juice, maceration, raw, tea	Oral	49.53
						Indigestion	Clear soup with flour dumplings, juice, tea	Oral	16.82
	*Sophora flavescens* Solander ex Aiton	KH5421	S180	Gosam	Leaf	Cattle disease	Infusion	Oral	61.96
					Root	Abdominal pain	Maceration	Oral	0.54
						Cattle disease	Infusion, juice, raw	Oral	61.96
						Chronic myofascial pain	Dissolution	Oral	2.17
						Gastroenteric disorder	Infusion	Oral	3.80
						Hansen's Disease	Dried, infusion	Oral	3.80
						Leg pain	Infusion, maceration	Oral	5.43
						Lumbago	Juice, maceration	Oral	7.61
						Neuralgia	Infusion, juice	Oral	5.43
						Paralysis	Infusion	Oral	2.72
						Pruritus	Dried, maceration, mixed in liquor, pill	Oral	1.63
						Pus	Maceration	Topical	2.72
						Skin diseases	Maceration, raw	Oral	2.17
	*Vigna angularis* (Willd.) Ohwi & H.Ohashi	KH5422	S190	Pat	Seed	Cattle disease	Infusion	Oral	100.00
Fagaceae	*Castanea crenata* Siebold & Zucc.	KH5423	S38	Bamnamu	Bark	Indigestion	Infusion	Oral	6.02
						Lacquer poisoning	Infusion	Topical	27.71
					Fruit	Abdominal cold hypersentivity	Decoction, grain syrup, infusion, pill, simmer	Oral	36.14
						Anorexia	Simmer, pill	Oral	6.02
						Lack of energy	Simmer, pill	Oral	6.02
						Measles	Panbroiled	Oral	3.61
						Sterility	Grain syrup, infusion, Simmer, pill	Oral	14.46
					Leaf	Lacquer poisoning	Infusion	Topical	27.71
					Stem	Lacquer poisoning	Infusion	Topical	27.71
	*Quercus acutissima* Carruth.	KH5424	S148	Sangsurinamu	Fruit	Cattle disease	Raw	Oral	100.00
Ganodermataceae	*Ganoderma lucidum* (Curtis) P. Karst.	KH5425	S77	Bulrocho	Whole part	Abdominal pain	Boiling	Oral	4.35
						Bone diseases	A sweet drink made from fermented rice	Oral	13.04
						Cancer	Boiling, decoction	Oral	26.09
						Common cold	Boiling	Oral	26.09
						Indigestion	Infusion	Oral	13.04
						Woman diseases	Dried, infusion	Oral	17.39
Gentianaceae	*Gentiana scabra* Bunge	KH5426	S80	Yongdam	Leaf	Pollakiuria	Infusion	Oral	45.45
					Root	Abdominal pain	A sweet drink made from fermented rice, infusion	Oral	54.55
Geraniaceae	*Geranium thunbergii* Siebold & Zucc.	KH5427	S81	Ijilpul	Leaf	Abdominal pain	Infusion	Oral	100.00
					Whole part	Abdominal pain	Infusion	Oral	100.00
Ginkgoaceae	*Ginkgo biloba* L.	KH5428	S82	Eunhaengnamu	Fruit	Asthma	Brewing	Oral	33.33
						Glycosuria	Infusion, roast	Oral	66.67
Hymenochaetaceae	*Phellinus linteus* (Berk. & Curt.) Teng	KH5429	S124	Mokjiljinheulkbeoseot	Whole part	Cancer	Boiling, infusion	Oral	68.97
						Gastroenteric disorder	Infusion	Oral	10.34
						Hypofunction	Infusion	Oral	3.45
						Lumbago	Infusion	Oral	17.24
Juglandaceae	*Juglans regia* L.	KH5430	S94	Hodunamu	Fruit	Lack of energy	Raw	Oral	100.00
	*Platycarya strobilacea* Siebold & Zucc.	KH5431	S133	Gulpinamu	Stem	Abdominal pain	Infusion	Oral	1.27
						Anorexia	Infusion	Oral	0.76
						Arm pain	A sweet drink made from fermented rice, brewing, decoction	Oral	6.36
						Bone diseases	A sweet drink made from fermented rice, brewing, decoction, infusion	Oral	60.31
						Cattle disease	Infusion	Oral	3.82
						Hyperthermia	A sweet drink made from fermented rice, brewing	Oral	0.51
						Leg pain	A sweet drink made from fermented rice, brewing, decoction, infusion	Oral	16.03
						Lumbago	A sweet drink made from fermented rice, infusion	Oral	10.43
						Neuralgia	Infusion	Oral	0.51
Lamiaceae	*Leonurus japonicus* Houtt.	KH5432	S100	Ikmocho	Aerial part	Abdominal cold hypersentivity	A sweet drink made from fermented rice, decoction, dried, grain syrup, infusion, juice, maceration, pill, raw, simmer, taffy	Oral	42.17
						Abdominal pain	Infusion, juice	Oral	2.07
						Afterpain	Maceration	Oral	1.61
						Anorexia	Maceration, pill, simmer	Oral	3.00
						Bone diseases	A sweet drink made from fermented rice, dried, infusion	Oral	0.92
						Cattle disease	Brewing, maceration	Oral	1.38
						Dysmenorrhea	Infusion	Oral	1.61
						Fatigue	Maceration	Oral	1.38
						Jaundice	A sweet drink made from fermented rice, infusion, juice, pill, tea	Oral	1.84
						Lack of energy	A sweet drink made from fermented rice, dried, infusion, pill, simmer	Oral	2.07
						Leukorrhea	Infusion	Oral	2.53
						Liver diseases	Infusion, pill	Oral	0.46
						Raynaud's Phenomenon	A sweet drink made from fermented rice, infusion, juice	Oral	4.84
						Sterility	A sweet drink made from fermented rice, decoction, infusion, juice, maceration, pill, simmer	Oral	21.20
						Woman diseases	Boiling dried, grain syrup, infusion, juice, pill, simmer, taffy	Oral	10.60
					Leaf	Abdominal cold hypersentivity	Grain syrup, infusion, pill	Oral	42.17
						Anorexia	Juice	Oral	3.00
						Irregular menstruation	Infusion	Oral	0.46
						Raynaud's Phenomenon	Infusion	Oral	4.84
						Sterility	Grain syrup, infusion, simmer	Oral	21.20
						Woman diseases	Grain syrup	Oral	10.60
					Root	Sterility	Infusion	Oral	21.20
					Stem	Raynaud's Phenomenon	Juice	Oral	4.84
						Sterility	Grain syrup, juice	Oral	21.20
					Whole part	Abdominal cold hypersentivity	Decoction, grain syrup, infusion, juice	Oral	42.17
						Paralysis	Decoction, juice	Oral	0.92
						Sterility	Grain syrup, juice	Oral	21.20
						Overheating	Juice	Oral	0.92
						Woman diseases	Decoction, juice	Oral	10.60
	*Mentha piperascens* (Malinv.) Holmes	KH5433	S107	Bakha	Stem	Dental pain	Maceration	Topical	100.00
	*Perilla frutescens* var. *acuta* Kudo	KH5434	S121	Soyeop	Leaf	Atopic dermatitis	Infusion, juice	Oral, topical	7.69
						Cattle disease	Infusion	Oral	25.00
						Common cold	Decoction, infusion	Oral	57.69
						Headache	Decoction	Oral	1.92
						Pruritus	Infusion, juice	Oral, topical	7.69
	*Perilla frutescens* var. *japonica* (Hassk.) H.Hara	KH5435	S122	Deulggae	Seed	Abdominal pain	Raw	Oral	13.73
						Convulsion	Decoction, oil	Oral	31.37
						Gastroenteric disorder	Seasoned cooked vegetables	Oral	7.84
						Lacquer poisoning	Maceration, powder, raw	Topical	47.06
	*Salvia plebeia* R.Br.	KH5436	S164	Baeamchajeugi	Leaf	Bone diseases	A sweet drink made from fermented rice, dried, infusion,	Oral	7.22
						Gastroenteric disorder	Infusion	Oral	5.15
						Hypofunction	A sweet drink made from fermented rice, dried, infusion,	Oral	10.31
					Root	Leg pain	A sweet drink made from fermented rice	Oral	19.59
					Stem	Arthritis	Infusion	Oral	3.09
					Whole part	Abdominal pain	Infusion	Oral	7.22
						Arm pain	A sweet drink made from fermented rice	Oral	5.15
						Asthma	A sweet drink made from fermented rice, dried, infusion,	Oral	4.12
						Bone diseases	Infusion	Oral	7.22
						Cough	Dried, grain syrup, infusion	Oral	28.87
						Empyema	Infusion	Oral	3.09
						Hypofunction	A sweet drink made from fermented rice, brewing	Oral	10.31
						Leg pain	A sweet drink made from fermented rice, infusion	Oral	19.59
						Woman diseases	Infusion	Oral	6.19
Lardizabalaceae	*Akebia quinata* (Houtt.) Decne.	KH5437	S7	Eureumdeonggul	Stem	Abdominal pain	Infusion	Oral	4.84
						Anorexia	Infusion	Oral	4.84
						Arm pain	A sweet drink made from fermented rice	Oral	6.45
						Bone diseases	A sweet drink made from fermented rice, infusion	Oral	46.77
						Leg pain	A sweet drink made from fermented rice	Oral	6.45
						Pollakiuria	Infusion	Oral	20.97
						Sterility	Infusion	Oral	9.68
Lauraceae	*Cinnamomum cassia* Presl	KH5438	S44	Gyepinamu	Bark	Common cold	Decoction	Oral	100.00
	*Lindera obtusiloba* Blume	KH5439	S101	Saenggangnamu	Stem	Arm pain	A sweet drink made from fermented rice	Oral	28.00
						Leg pain	Brewing	Oral	28.00
						Lumbago	Infusion	Oral	28.00
						Postpartum myofascial pain syndrome	Infusion	Oral	16.00
Liliaceae	*Allium fistulosum* L.	KH5440	S9	Pa	Root	Common cold	Decoction, infusion	Oral	65.52
						Cough	Infusion	Oral	12.07
						Hyperthermia	Infusion	Oral	22.41
	*Allium scorodorpasum* var. *viviparum* Regel	KH5441	S10	Maneul	Bulb	Lymphnoditis	Maceration	Topical	100.00
	*Allium thunbergii* G.Don	KH5442	S11	Sanbuchu	Aerial part	Angina pectoris	Kimchi, raw, seasoned cooked vegetables	Oral	100.00
	*Allium tuberosum* Rottler ex Spreng.	KH5443	S12	Buchu	Leaf	Epistaxis	Juice	Oral	18.18
						Lacquer poisoning	Maceration, raw	Topical	48.48
					Leaf	Pruritus	Maceration	Topical	12.12
					Root	Pollakiuria	Infusion, juice	Oral	21.21
	*Asparagus schoberioides* Kunth	KH5444	S25	Bijjaru	Whole part	Edema	Infusion	Oral	13.46
						Pollakiuria	A sweet drink made from fermented rice, infusion	Oral	73.08
						Sexual enhancement	Infusion	Oral	13.46
	*Scilla scilloides* (Lindl.) Druce	KH5445	S170	Mureut	Root	Edema	Simmer	Oral	100.00
Loranthaceae	*Viscum album* var. *coloratum* (Kom.) Ohwi	KH5446	S193	Gyeousali	Leaf	Edema	A sweet drink made from fermented rice	Oral	100.00
					Stem	Edema	A sweet drink made from fermented rice	Oral	100.00
Magnoliaceae	*Magnolia obovata* Thunb.	KH5447	S106	Ilbonmokryeon	Fruit	Heart disease	Infusion	Oral	100.00
Malvaceae	*Althaea rosea* (L.) Cav.	KH5448	S13	Jeopsikkot	Root	Leukorrhea	Infusion	Oral	50.00
						Sterility	Infusion	Oral	50.00
					Whole part	Sterility	Decoction	Oral	50.00
Meliaceae	*Cedrela sinensis* Juss.	KH5449	S40	Chamjuknamu	Flower	Sterility	Simmer	Oral	100.00
Menispermaceae	*Cocculus trilobus* (Thunb.) DC.	KH5450	S49	Daengdaengideonggul	Fruit	Cough	Mixed in ripe persimmon, raw	Oral	35.29
					Leaf	Food poisoning	Infusion	Oral	11.76
					Root	Acute gastroenteritis	Infusion, maceration	Oral	47.06
						Indigestion	Juice	Topical	5.88
					Stem	Acute gastroenteritis	Infusion	Oral	47.06
						Food poisoning	Infusion	Oral	11.76
Moraceae	*Broussonetia kazinoki* Siebold	KH5451	S31	Daknamu	Fruit	Hookworm	Infusion	Oral	100.00
	*Cudrania tricuspidata* (Carr.) Bureau ex Lavallee	KH5452	S59	Kkujippongnamu	Bark	Cancer	Infusion	Oral	25.31
						Glycosuria	Infusion	Oral	33.44
						Liver diseases	Infusion	Oral	6.56
					Leaf	Extravasated blood	Dried, maceration	Oral	1.25
					Root	Bone diseases	A sweet drink made from fermented rice	Oral	17.19
						Cancer	A sweet drink made from fermented rice, boiling, brewing, infusion	Oral	25.31
						Poor circulation	Infusion	Oral	1.88
						Glycosuria	A sweet drink made from fermented rice, brewing, decoction, infusion	Oral	33.44
						Hypofunction	A sweet drink made from fermented rice, infusion	Oral	7.50
						Leg pain	Infusion	Oral	1.25
						Lumbago	Infusion	Oral	3.75
						Neuralgia	Infusion	Oral	1.25
						Paralysis	Infusion	Oral	0.63
					Stem	Bone diseases	A sweet drink made from fermented rice, brewing, infusion	Oral	17.19
						Cancer	A sweet drink made from fermented rice, boiling, brewing, infusion	Oral	25.31
						Poor circulation	Infusion	Oral	1.88
						Glycosuria	A sweet drink made from fermented rice, brewing, infusion	Oral	33.44
						Hypofunction	A sweet drink made from fermented rice, infusion	Oral	7.50
						Leg pain	Infusion	Oral	1.25
						Liver diseases	A sweet drink made from fermented rice, infusion	Oral	6.56
						Lumbago	A sweet drink made from fermented rice, infusion	Oral	3.75
						Paralysis	Infusion	Oral	0.63
	*Morus alba* L.	KH5453	S108	Ppongnamu	Stem	Cancer	Dried, infusion	Oral	33.33
						Glycosuria	Infusion	Oral	66.67
					Young leaf	Cancer	Dried, infusion	Oral	33.33
						Glycosuria	Dried, infusion, panbroiled, raw, tea	Oral	66.67
Nymphaeaceae	*Nelumbo nucifera* Gaertn.	KH5454	S109	Yeonggot	Root	Epistaxis	Infusion, juice	Oral	100.00
Oleaceae	*Forsythia koreana* (Rehder) Nakai	KH5455	S76	Gaenari	Stem	Otalgia	Infusion	Topical	100.00
Orchidaceae	*Gastrodia elata* Blume	KH5456	S79	Cheonma	Root	Lack of energy	Brewing	Oral	100.00
Paeoniaceae	*Paeonia obovata* Maxim.	KH5457	S116	Sanjakyak	Root	Irregular menstruation	Infusion	Oral	37.50
						Lack of energy	Boiling, decoction	Oral, topical	62.50
Papaveraceae	*Chelidonium majus* var. *asiaticum* (H.Hara) Ohwi	KH5458	S43	Aegittongpul	Aerial part	Bone diseases	A sweet drink made from fermented rice, dried, infusion,	Oral	5.06
						Chronic myofascial pain	Juice	Oral	68.35
						Hypofunction	A sweet drink made from fermented rice, dried, infusion,	Oral	5.06
					Root	Chronic myofascial pain	Brewing	Oral	68.35
					Stem	Chronic myofascial pain	Brewing	Oral	68.35
					Whole part	Chronic myofascial pain	A sweet drink made from fermented rice, brewing, dissolution, infusion, juice, maceration	Oral, topical	68.35
						Glycosuria	Brewing	Oral	5.06
						Induced abortion	Juice	Oral	11.39
						Lumbago	Brewing	Oral	5.06
	*Papaver somniferum* L.	KH5459	S117	Yanggwibi	Fruit	Abdominal pain	Dissolution, dried, grain syrup, juice, maceration, raw	Oral	53.76
						Bone diseases	Infusion	Oral	6.45
						Cattle disease	Dried, dissolution, infusion	Oral	15.05
						Diarrhea	Dried, dissolution	Oral	2.15
						Hypofunction	Infusion	Oral	8.60
						Pain	Dried, dissolution	Oral	3.23
						Sinews and joint pain	Dissolution	Oral	3.23
						Wound	Dried, dissolution, raw	Topical	3.23
					Leaf	Abdominal pain	Raw	Oral	53.76
					Stem	Abdominal pain	Dried, infusion	Oral	53.76
						Bone diseases	Infusion	Oral	6.45
						Cattle disease	Infusion	Oral	15.05
						Hoove	Infusion	Oral	4.30
					Whole part	Abdominal pain	Brewing	Oral	53.76
Pedaliaceae	*Sesamum indicum* L.	KH5460	S173	Chamggae	Seed	Abdominal cold hypersentivity	Simmer	Oral	21.05
						Burn	Oil	Topical	21.05
						Convulsion	Oil	Oral	10.53
						Hemorrhaging	Raw, rubbing	Topical	21.05
						Otalgia	Oil	Topical	26.32
Phytolaccaceae	*Phytolacca esculenta* VanHoutte	KH5461	S128	Jarigong	Root	Abdominal pain	Infusion	Oral	10.42
						Cattle disease	Juice	Oral	8.33
						Constipation	Raw	Topical	12.50
						Edema	A sweet drink made from fermented rice, infusion	Oral	39.58
						Indigestion	Infusion	Oral	4.17
						Lumbago	A sweet drink made from fermented rice, brewing, infusion	Oral	25.00
Pinaceae	*Pinus densiflora* Siebold & Zucc.	KH5462	S130	Sonamu	Leaf	Dental pain	Raw	Topical	33.33
						Lumbago	Steamed	Topical	11.11
					Pollen	Hemorrhaging	Powder	Topical	22.22
					Stem	Otalgia	Infusion	Topical	33.33
	*Pinus koraiensis* Siebold & Zucc.	KH5463	S131	Jatnamu	Pine resin	Boils	Raw	Topical	44.44
						Wound	Melt	Topical	55.56
Plantaginaceae	*Plantago asiatica* L.	KH5464	S132	Jilgyeongi	Leaf	Heart disease	Infusion	Oral	4.97
					Root	Abdominal pain	Juice	Oral	0.62
						Pollakiuria	Infusion	Oral	78.26
					Whole part	Epistaxis	Dried,infusion, raw	Oral	8.70
						Gastroenteric disorder	Decoction	Oral	3.73
						Heart disease	Infusion	Oral	4.97
						Pollakiuria	Decoction, dried, infusion, raw	Oral	78.26
						Sterility	Infusion	Oral	3.73
Poaceae	*Hordeum vulgare* var. *hexastichon* (L.) Asch.	KH5465	S87	Bori	Malt	Abdominal pain	Malted barley seedling	Oral	0.62
						Arm pain	A sweet drink made from fermented rice, infusion	Oral	6.69
						Arthritis	A sweet drink made from fermented rice, infusion	Oral	1.87
						Bone diseases	A sweet drink made from fermented rice, brewing, dried, infusion	Oral	29.70
						Cancer	A sweet drink made from fermented rice	Oral	0.47
						Carpal tunnel syndrome	A sweet drink made from fermented rice	Oral	1.09
						Chronic myofascial pain	A sweet drink made from fermented rice	Oral	1.09
						Common cold	Decoction	Oral	0.78
						Cough	A sweet drink made from fermented rice	Oral	1.87
						Edema	A sweet drink made from fermented rice	Oral	1.87
						Eye disease	Boiling, dried	Oral	0.78
						Fatigue	A sweet drink made from fermented rice	Oral	1.09
						Finger pain	Decoction	Oral	1.24
						Gastric ulcer	A sweet drink made from fermented rice	Oral	0.16
						Gastritis	A sweet drink made from fermented rice	Oral	0.16
						Gastroenteric disorder	A sweet drink made from fermented rice, infusion, pill	Oral	3.73
						Hemorrhaging	Rubbing	Topical	0.78
						Hookworm	A sweet drink made from fermented rice	Oral	0.47
						Hyperthermia	A sweet drink made from fermented rice	Oral	0.16
						Hypofunction	A sweet drink made from fermented rice, dried, infusion	Oral	1.87
						Indigestion	A sweet drink made from fermented rice, infusion, maceration, malted barley seedling, raw	Oral	4.98
						Jaundice	A sweet drink made from fermented rice	Oral	2.33
						Leg pain	A sweet drink made from fermented rice, decoction, infusion	Oral	14.46
						Liver diseases	A sweet drink made from fermented rice, pill	Oral	1.24
						Lumbago	A sweet drink made from fermented rice, brewing	Oral	6.22
						Paralysis	A sweet drink made from fermented rice, infusion	Oral	4.82
						Pollakiuria	A sweet drink made from fermented rice	Oral	1.09
						Pruritus	A sweet drink made from fermented rice	Oral	1.71
						Pus	Boiled rice, maceration	Topical	1.87
						Raynaud's Phenomenon	A sweet drink made from fermented rice	Oral	0.31
						Sexual enhancement	A sweet drink made from fermented rice	Oral	0.31
						Shoulder pain	A sweet drink made from fermented rice	Oral	1.56
						Sinews and joint pain	A sweet drink made from fermented rice	Oral	0.16
						Skin diseases	A sweet drink made from fermented rice	Oral	0.93
						Tarsal tunnel syndrome	A sweet drink made from fermented rice	Oral	1.09
						Woman diseases	Grain syrup	Oral	0.47
	*Hordeum vulgare* var. *nudum* Spenn.	KH5466	S88	Ssalbori	Seed	Pollakiuria	A sweet drink made from fermented rice	Oral	100.00
	*Imperata cylindrica* var. *koenigii* (Retz.) Pilg.	KH5467	S92	Tti	Flower	Bloody discharge	Infusion	Oral	15.38
						Burn	Burn, raw	Topical	84.62
	*Oryza sativa* L.	KH5468	S113	Byeo	Root	Lacquer poisoning	Infusion	Topical	48.98
					Seed	Carpal tunnel syndrome	Infusion	Oral	4.08
						Hangover	Powder	Oral	4.08
						Jaundice	A sweet drink made from fermented rice	Oral	5.10
						Lacquer poisoning	Maceration, raw	Topical	48.98
						Pollakiuria	Infusion, rice water	Oral	9.18
						Snakebite	Porridge, raw	Topical	8.16
						Tarsal tunnel syndrome	Infusion	Oral	4.08
					Stem	Burn	Raw	Topical	2.04
						Hemorrhaging	Raw	Topical	2.04
						Indigestion	Dried, infusion	Oral	2.04
						Urticaria	Roast	Topical	5.10
						Wound	Raw	Topical	5.10
	*Oryza sativa* var. *glutinosa* Blanco	KH5469	S114	Chalbyeo	Seed	Abdominal cold hypersentivity	Pill, simmer	Oral	31.25
						Anorexia	Pill, simmer	Oral	7.81
						Carpal tunnel syndrome	A sweet drink made from fermented rice	Oral	3.13
						Lack of energy	Pill, simmer	Oral	7.81
						Paralysis	A sweet drink made from fermented rice	Oral	12.50
						Pollakiuria	Porridge	Oral	7.81
						Sterility	Pill, simmer	Oral	7.81
						Tarsal tunnel syndrome	A sweet drink made from fermented rice	Oral	3.13
					Stem	Cattle disease	Fumigation	Topical	7.81
						Urticaria	Fumigation	Topical	7.81
					Young leaf	Epistaxis	Infusion	Oral	3.13
	*Phyllostachys bambusoides* Siebold & Zucc.	KH5470	S126	Wangdae	Leaf	Common cold	Infusion	Oral	38.46
					Root	Lumbago	A sweet drink made from fermented rice	Oral	61.54
	*Sasa borealis* (Hack.) Makino	KH5471	S167	Joritdae	Aerial part	Cattle disease	Infusion	Oral	100.00
	*Sasa coreana* Nakai	KH5472	S168	Sinidae	Leaf	Common cold	Decoction, infusion	Oral	56.25
					Stem	Cattle disease	Infusion	Oral	43.75
	*Setaria italica* (L.) P.Beauv.	KH5473	S174	Jo	Aerial part	Cattle disease	Infusion	Oral	100.00
	*Triticum aestivum* L.	KH5474	S187	Mil	Seed	Arm pain	A sweet drink made from fermented rice, brewing	Oral	5.26
						Asthma	Brewing	Oral	0.58
						Boils	Dough	Topical	2.05
						Bone diseases	A sweet drink made from fermented rice, brewing, dough, infusion	Oral, topical	19.30
						Bruising	Dough	Topical	9.94
						Carpal tunnel syndrome	A sweet drink made from fermented rice, brewing, dough	Oral, topical	2.05
						Cattle disease	Brewing	Oral	0.88
						Chronic myofascial pain	Brewing, chopped noodles, clear soup with flour dumplings, dissolution, dried, mixed in liquor, powder, roast	Oral	9.94
						Common cold	Brewing, tea	Oral	2.92
						Cough	Brewing	Oral	1.46
						Extravasated blood	Dough	Topical	0.29
						Gastroenteric disorder	A sweet drink made from fermented rice, brewing, juice	Oral	5.56
						Hangover	Clear soup with flour dumplings	Oral	1.75
						Hookworm	Infusion	Oral	2.05
						Hyperthermia	Brewing	Oral	0.29
						Indigestion	Clear soup with flour dumplings, extraction	Oral	2.92
						Leg pain	A sweet drink made from fermented rice, brewing	Oral	12.28
						Lumbago	Brewing, powder	Oral	8.48
						Milk fever	Clear soup with flour dumplings	Oral	2.05
						Orchiopathy	Brewing	Oral	0.58
						Paralysis	Brewing	Oral	1.17
						Pus	Dough, powder	Topical	2.92
						Sprain	Dough	Topical	2.63
						Tarsal tunnel syndrome	A sweet drink made from fermented rice, brewing, dough	Oral, topical	2.63
	*Zea mays* L.	KH5475	S198	Oksusu	Fruit	Pollakiuria	Tea	Oral	94.62
					Style	Abdominal pain	Infusion	Oral	2.31
						Anorexia	Infusion	Oral	2.31
						Lung diseases	Infusion	Oral	0.77
						Pollakiuria	Decoction, dried, infusion	Oral	94.62
Polygonaceae	*Fagopyrum esculentum* Moench	KH5476	S72	Memil	Pericarp	Pus	Maceration	Topical	100.00
	*Fallopia japonica* (Houtt.) RonseDecr.	KH5477	S73	Hojanggeun	Aerial part	Pollakiuria	Infusion	Oral	100.00
					Root	Pollakiuria	A sweet drink made from fermented rice, infusion	Oral	100.00
					Whole part	Pollakiuria	Infusion	Oral	100.00
	*Fallopia multiflora* (Thunb.) Haraldson	KH5478	S74	Hasuo	Root	Lack of energy	Dried, powder	Oral	9.09
						Sthenia	Brewing	Oral, topical	90.91
	*Rumex acetosa* L.	KH5479	S160	Suyeong	Whole part	Pollakiuria	Infusion	Oral	41.18
						Prostate disease	Infusion	Oral	41.18
						Renal disease	Infusion	Oral	17.65
	*Rumex crispus* L.	KH5480	S161	Sorijaengi	Root	Leg pain	A sweet drink made from fermented rice	Oral	38.89
						Malaria	Maceration	Oral	27.78
					Whole part	Leg pain	Brewing	Oral	38.89
						Lumbago	Brewing	Oral	33.33
Polyporaceae	*Coriolus versicolor* (L.: Fr.) Quél.	KH5481	S54	Gureumbeoseot	Whole part	Common cold	Boiling, decoction	Oral	100.00
	*Poria cocos* Wolf	KH5482	S137	Bokryeong	Whole part	Hoove	Powder	Oral	100.00
Portulacaceae	*Portulaca oleracea* L.	KH5483	S138	Soebireum	Aerial part	Cancer	Infusion	Oral	83.33
						Diarrhea	Seasoned cooked vegetables	Oral	16.67
Punicaceae	*Punica granatum* L.	KH5484	S145	Seokryunamu	Flower	Epistaxis	Dried, infusion	Topical	20.69
					Fruit	Abdominal pain	Raw	Oral	13.79
						Common cold	Extraction, infusion, raw	Oral	41.38
						Cough	Infusion	Oral	24.14
					Pericarp	Epistaxis	Dried	Topical	20.69
Ramariaceae	*Ramaria botrytis* (Pers.) Ricken	KH5485	S149	Ssaribeoseot	Whole part	Diarrhea	Infusion	Oral	100.00
Ranunculaceae	*Aconitum ciliare* DC.	KH5486	S3	Notjeotgaraknamul	Root	Afterpain	Infusion	Oral	1.08
						Arm pain	A sweet drink made from fermented rice, infusion	Oral	8.92
						Bone diseases	A sweet drink made from fermented rice, infusion	Oral	6.22
						Carpal tunnel syndrome	A sweet drink made from fermented rice, decoction, infusion, simmer	Oral	15.41
						Leg pain	A sweet drink made from fermented rice, decoction, infusion	Oral	14.05
						Lumbago	Infusion	Oral	0.81
						Malgia	Infusion	Oral	0.27
						Paralysis	A sweet drink made from fermented rice, brewing, decoction, infusion, simmer	Oral	37.84
						Postpartum myofascial pain syndrome	Infusion	Oral	1.08
						Tarsal tunnel syndrome	A sweet drink made from fermented rice, decoction, infusion, simmer	Oral	14.32
	*Aconitum coreanum* (H.Lév.) Rapaics	KH5487	S4	Baekbuja	Stem	Bone diseases	Infusion	Oral	50.00
						Sinews and joint pain	Infusion	Oral	50.00
	*Pulsatilla koreana* (Yabe ex Nakai) Nakai ex Nakai	KH5488	S144	Halmikkot	Leaf	Facial nerve paralysis	Maceration	Topical	2.99
					Root	Abdominal pain	A sweet drink made from fermented rice, grain syrup, infusion	Oral	11.19
						Bone diseases	A sweet drink made from fermented rice, brewing	Oral	2.99
						Convulsion	Infusion	Oral	2.99
						Facial nerve paralysis	Maceration	Topical	2.99
						Gastroenteric disorder	A sweet drink made from fermented rice, infusion, simmer	Oral	8.96
						Hookworm	A sweet drink made from fermented rice, decoction, dissolution, grain syrup, infusion, juice, maceration	Oral, topical	60.45
						Hypoacusis	Brewing	Oral	3.73
						Lumbago	A sweet drink made from fermented rice, brewing	Oral	2.99
						Lymphnoditis	Maceration	Topical	3.73
	*Ranunculus sceleratus* L.	KH5489	S150	Gaegurijari	Leaf	Callus	Maceration	Topical	12.07
						Facial nerve paralysis	Maceration, raw	Topical	87.93
					Root	Facial nerve paralysis	Maceration	Topical	87.93
Rhamnaceae	*Hovenia dulcis* Thunb.	KH5490	S89	Heotgaenamu	Fruit	Cancer	Decoction	Oral	5.56
						Liver diseases	Tea	Oral	69.44
					Stem	Cancer	Decoction	Oral	5.56
						Glycosuria	Infusion	Oral	13.89
						Hangover	Brewing, infusion	Oral	11.11
						Liver diseases	Infusion	Oral	69.44
	*Zizyphus jujuba* var. *inermis* (Bunge) Rehder	KH5491	S200	Daechunamu	Fruit	Abdominal cold hypersentivity	Decoction, grain syrup, infusion, pill, simmer	Oral	14.29
						Anorexia	Pill, simmer	Oral	2.38
						Common cold	Boiling, decoction, dried, roast	Oral	2.38
						Cough	Infusion	Oral	8.10
						Fatigue	A sweet drink made from fermented rice, boiled rice, raw	Oral	10.00
						Gastroenteric disorder	Infusion	Oral	0.95
						Lack of energy	Decoction, pill, simmer, tea	Oral	3.33
						Sterility	Grain syrup, infusion, pill, simmer	Oral	8.10
						Woman diseases	Infusion	Oral	3.81
					Leaf	Overheating	Raw	Topical	0.48
					Root	Sterility	Infusion	Oral	8.10
					Stem	Arm pain	Infusion	Oral	0.95
						Facial nerve paralysis	Raw	Topical	44.29
						Leg pain	Infusion	Oral	0.95
Rosaceae	*Chaenomeles sinensis* (Thouin) Koehne	KH5492	S42	Mogwanamu	Fruit	Common cold	Boiling, brewing, decoction, extraction, infusion	Oral	45.36
						Cough	Boiling, brewing, decoction, dissolution, extraction, infusion, steamed	Oral	54.64
	*Duchesnea indica* (Andr.) Focke	KH5493	S64	Baemddalgi	Fruit	Dental pain	Infusion	Oral	46.15
						Snakebite	Maceration	Topical	38.46
					Whole part	Dental pain	Infusion	Topical	46.15
						Measles	Infusion	Oral	15.38
	*Potentilla chinensis* Ser.	KH5494	S139	Ttakjikkoch	Root	Gastroenteric disorder	Infusion, raw	Oral	100.00
	*Prunus armeniaca* var. *ansu* Maxim.	KH5495	S140	Salgunamu	Seed	Indigestion	Maceration	Oral	100.00
	*Prunus davidiana* (Carrière) Franch.	KH5496	S141	Sanboksanamu	Fruit	Abdominal pain	Extraction	Oral	6.15
						Arm pain	Brewing, extraction	Oral	21.54
						Hypertension	Brewing	Oral	4.62
						Leg pain	Brewing, extraction	Oral	44.62
						Lumbago	Brewing	Oral	9.23
						Osteoporosis	Extraction	Oral	1.54
					Root	Leg pain	Brewing	Oral	44.62
						Lumbago	Brewing	Oral	9.23
					Stem	Asthma	Grain syrup	Oral	4.62
						Bone diseases	Infusion	Oral	7.69
						Leg pain	Infusion	Oral	44.62
	*Prunus mume* Siebold & Zucc.	KH5497	S142	Maesilnamu	Fruit	Abdominal pain	Extraction	Oral	46.00
						Gastroenteric disorder	Extraction	Oral	10.00
						Indigestion	Brewing, extraction	Oral	44.00
	*Pyrus pyrifolia* (Burm.f.) Nakai	KH5498	S146	Dolbaenamu	Fruit	Asthma	Fermentation	Oral	40.00
						Cough	Fermentation	Oral	40.00
						Leg pain	Brewing, raw	Oral	20.00
	*Pyrus pyrifolia* var. *culta* (Makino) Nakai	KH5499	S147	Baenamu	Fruit	Bronchitis	Extraction, infusion	Oral	5.88
						Common cold	A sweet drink made from fermented rice, decoction, fermentation, infusion, raw, roast	Oral	56.86
						Cough	Infusion, raw, roast, steamed	Oral	33.33
						Indigestion	Infusion	Oral	3.92
	*Rosa multiflora* Thunb.	KH5500	S158	Jjilrekkot	Root	Osteoporosis	Infusion	Oral	100.00
	*Rubus coreanus* Miq.	KH5501	S159	Bokbunjaddalgi	Fruit	Sexual enhancement	Extraction, raw	Oral	100.00
	*Sanguisorba officinalis* L.	KH5502	S165	Oipul	Leaf	Cattle disease	Juice	Oral	7.69
						Hoove	Juice	Oral	7.69
					Root	Cattle disease	Juice	Oral	7.69
						Finger pain	Infusion	Topical	3.85
						Hoove	Juice	Oral	7.69
						Hypofunction	Decoction, simmer	Oral, topical	7.69
						Leg pain	Infusion	Topical	23.08
						Pus	Infusion	Topical	3.85
						Sprain	Infusion	Topical	19.23
						Toe pain	Infusion	Topical	3.85
					Whole part	Dysentery	Infusion	Oral	23.08
	*Sorbus commixta* Hedl.	KH5503	S181	Magamok	Stem	Arm pain	A sweet drink made from fermented rice	Oral	25.00
						Leg pain	A sweet drink made from fermented rice	Oral	25.00
						Shoulder pain	A sweet drink made from fermented rice	Oral	50.00
	*Spiraea prunifolia* f. *simpliciflora* Nakai	KH5504	S182	Jopapnamu	Root	Indigestion	Infusion	Oral	94.12
					Stem	Indigestion	Infusion	Oral	94.12
						Leg pain	Infusion	Oral	5.88
Rubiaceae	*Gardenia jasminoides* J.Ellis	KH5505	S78	Chijanamu	Fruit	Bone diseases	Dough	Topical	1.43
						Bruising	Dough, infusion	Topical	61.43
						Carpal tunnel syndrome	Infusion	Topical	2.86
						Chronic myofascial pain	Infusion	Topical	4.29
						Extravasated blood	Dough	Topical	1.43
						Pus	Brewing	Oral	5.71
						Sprain	Dough, infusion	Topical	20.00
						Tarsal tunnel syndrome	Infusion	Topical	2.86
Rutaceae	*Citrus unshiu* S.Marcov.	KH5506	S47	Gyul	Pericarp	Common cold	Infusion	Oral	100.00
	*Phellodendron amurense* Rupr.	KH5507	S125	Hwangbyeoknamu	Bark	Arm pain	A sweet drink made from fermented rice	Oral	4.12
						Bone diseases	Brewing	Oral	61.42
						Hyperthermia	Brewing	Oral	0.37
						Indigestion	Infusion	Oral	3.75
						Leg pain	A sweet drink made from fermented rice, infusion	Oral	18.35
						Lumbago	A sweet drink made from fermented rice, infusion	Oral	11.24
					Endodermis	Indigestion	Steep	Oral	3.75
						Lumbago	Brewing	Oral	11.24
					Stem	Arm pain	A sweet drink made from fermented rice	Oral	4.12
						Bone diseases	A sweet drink made from fermented rice, brewing, infusion	Oral	61.42
						Leg pain	A sweet drink made from fermented rice, infusion	Oral	18.35
						Lumbago	A sweet drink made from fermented rice, infusion	Oral	11.24
						Neuralgia	Infusion	Oral	0.75
	*Poncirus trifoliata* (L.) Raf.	KH5508	S135	Taengjanamu	Fruit	Cough	Dried, infusion	Oral	14.00
						Lacquer poisoning	Infusion	Topical	12.00
						Urticaria	Infusion	Oral, topical	66.00
					Leaf	Snakebite	Maceration	Topical	8.00
	*Zanthoxylum piperitum* (L.) DC.	KH5509	S196	Chopinamu	Fruit	Asthma	Oil	Oral	0.32
						Carpal tunnel syndrome	Brewing, infusion	Oral	4.05
						Dental pain	Infusion, raw	Topical	1.78
						Gastroenteric disorder	Oil	Oral	0.32
						Leg pain	Brewing	Oral	14.08
						Paralysis	A sweet drink made from fermented rice, infusion, oil, simmer	Oral	25.24
						Tarsal tunnel syndrome	Brewing, infusion	Oral	4.05
					Pericarp	Carpal tunnel syndrome	Infusion	Oral	4.05
						Eye disease	Infusion	Oral	0.32
						Facial nerve paralysis	Infusion	Oral	1.62
						Headache	Infusion	Oral	0.65
						Hypertension	Powder	Oral	1.29
						Lumbago	Infusion	Oral	7.93
						Paralysis	A sweet drink made from fermented rice, infusion, oil, pan fried, simmer	Oral	25.24
						Tarsal tunnel syndrome	Infusion	Oral	4.05
					Root	Paralysis	Infusion	Oral	25.24
					Stem	Arm pain	A sweet drink made from fermented rice, brewing	Oral	6.31
						Bone diseases	A sweet drink made from fermented rice, brewing, decoction, infusion	Oral	31.23
						Carpal tunnel syndrome	A sweet drink made from fermented rice, infusion	Oral	4.05
						Common cold	Infusion	Oral	0.32
						Facial nerve paralysis	Infusion	Oral	1.62
						Hyperthermia	Brewing	Oral	0.16
						Leg pain	A sweet drink made from fermented rice, brewing, infusion	Oral	14.08
						Lumbago	A sweet drink made from fermented rice, brewing, infusion	Oral	7.93
						Neuralgia	Infusion	Oral	0.32
						Paralysis	A sweet drink made from fermented rice, infusion, oil, pan fried, simmer	Oral	25.24
						Tarsal tunnel syndrome	A sweet drink made from fermented rice, infusion	Oral	4.05
	*Zanthoxylum schinifolium* Siebold & Zucc.	KH5510	S197	Sanchonamu	Fruit	Abdominal pain	Oil	Oral	2.13
						Arthritis	Infusion	Oral	1.42
						Asthma	Oil, roast	Oral	8.87
						Boils	Oil	Oral, topical	9.22
						Bronchitis	Oil, pan fried	Oral	3.55
						Carpal tunnel syndrome	Brewing	Oral	0.35
						Cattle disease	Oil	Oral	12.41
						Cattle ringworm	Oil	Topical	1.42
						Common cold	Oil	Oral	0.35
						Constipation	Oil	Oral	1.42
						Convulsion	Oil	Oral	3.55
						Cough	Extraction, oil, pan fried, roast	Oral	20.92
						Dental pain	Raw	Topical	1.77
						Diarrhea	Roast	Oral	1.77
						Edema	Oil	Oral, topical	2.84
						Erysipelas	Oil, pan fried	Oral, topical	4.96
						Hoove	Oil	Oral	1.42
						Hypofunction	Oil	Oral	2.48
						Indigestion	Oil	Oral	0.71
						Measles	Oil	Oral	1.06
						Paralysis	Oil	Oral	3.19
						Pig disease	Oil	Oral	2.84
						Pus	Oil	Topical	1.77
						Stomatitis	Oil	Topical	1.77
						Tarsal tunnel syndrome	Brewing	Oral	1.06
						Whooping cough	Oil, pan fried	Oral	1.42
					Leaf	Snakebite	Rubbing	Topical	3.90
					Pine resin	Cattle disease	Dried, powder	Topical	12.41
					Stem	Bone diseases	Brewing	Oral	1.42
						Snakebite	Fumigation	Topical	3.90
Salicaceae	*Populus maximowiczii* A.Henry	KH5511	S136	Hwangcheolnamu	Endodermis	Gastroenteric disorder	Infusion	Oral	50.00
						Leg pain	Infusion	Oral	50.00
	*Salix gracilistyla* Miq.	KH5512	S162	Gaetbeodeul	Stem	Allergic contact dermatitis	Fumigation	Topical	33.33
						Lacquer poisoning	Fumigation	Topical	33.33
						Pruritus	Fumigation	Topical	33.33
	*Salix koreensis* Andersson	KH5513	S163	Beodeunamu	Bark	Fracture	Raw	Topical	60.00
					Fruit	Lacquer poisoning	Fumigation	Topical	33.33
					Stem	Facial nerve paralysis	Raw	Topical	6.67
						Fracture	Raw	Topical	60.00
Schisandraceae	*Schisandra chinensis* (Turcz.) Baill.	KH5514	S169	Omija	Fruit	Arm pain	Decoction, dissolution, extraction	, Oral	4.86
						Bronchitis	Extraction, infusion	Oral	5.56
						Poor circulation	Decoction	Oral	0.69
						Common cold	A sweet drink made from fermented rice, boiling, brewing, decoction, dissolution, dried, extraction, raw, tea	Oral	45.83
						Cough	Brewing, decoction, dissolution, extraction	Oral	15.97
						Eye disease	Boiling, dried, extraction	Oral	6.25
						Headache	Boiling, dried	Oral	3.47
						Heart disease	Dried, extraction, tea	Oral	5.56
						Lack of energy	Extraction	Oral	2.78
						Leg pain	Decoction, dissolution, extraction	Oral	4.86
					Stem	Abdominal pain	Infusion	Oral	2.08
						Anorexia	Infusion	Oral	2.08
Scrophulariaceae	*Paulownia coreana* Uyeki	KH5515	S120	Odongnamu	Fruit	Edema	Infusion	Oral	36.36
					Stem	Indigestion	Infusion	Oral	63.64
	*Rehmannia glutinosa* (Gaertn.) Libosch. ex Steud.	KH5516	S152	Jihwang	Root	Abdominal cold hypersentivity	Pill	Oral	1.21
						Arm pain	Maceration	Topical	3.64
						Bruising	Brewing, juice, maceration, mixed in liquor, raw	Oral, topical	29.70
						Carpal tunnel syndrome	Maceration	Topical	1.21
						Chronic myofascial pain	Dissolution, maceration	Oral, topical	6.06
						Edema	Maceration, raw	Oral	1.21
						Fracture	Juice, maceration	Oral, topical	15.15
						Lack of energy	Boiling, decoction	Oral	3.03
						Leg pain	Maceration	Topical	3.64
						Lumbago	Brewing, juice, maceration	Oral, topical	14.55
						Luxation	Maceration	Topical	4.24
						Sprain	Juice, maceration	Oral, topical	12.73
						Tarsal tunnel syndrome	Maceration	Topical	1.21
						Wound	Maceration	Topical	2.42
Selaginellaceae	*Selaginella tamariscina* (P.Beauv.) Spring	KH5517	S172	Bucheoson	Whole part	Bruising	Maceration	Topical	26.32
						Edema	Maceration	Topical	26.32
						Luxation	Maceration	Topical	26.32
						Sterility	Infusion	Oral	21.05
Simaroubaceae	*Picrasma quassioides* (D.Don) Benn.	KH5518	S129	Sotaenamu	Bark	Deprived of mother's milk	Maceration	Topical	32.88
						Lumbago	Infusion	Oral	10.96
					Leaf	Cattle disease	Infusion	Oral	19.18
					Stem	Arthritis	Simmer	Oral	9.59
						Boils	Oil	Topical	5.48
						Cattle disease	Infusion	Oral	19.18
						Chronic myofascial pain	Brewing	Oral	5.48
						Deprived of mother's milk	Infusion, juice, maceration	Topical	32.88
						Hoove	Infusion	Oral	6.85
						Lumbago	Infusion	Oral	10.96
						Pruritus	Infusion	Topical	9.59
Smilacaceae	*Smilax china* L.	KH5519	S176	Cheongmiraedeonggul	Rhizome	Hypofunction	Infusion	Topical	15.00
						Leg pain	Infusion	Oral	30.00
						Osteoporosis	Infusion	Oral	15.00
						Pollakiuria	Infusion	Oral	25.00
						Wound	Infusion	Topical	15.00
Solanaceae	*Capsicum annuum* L.	KH5520	S35	Gochu	Fruit	Chronic myofascial pain	Dissolution	Oral	100.00
	*Lycium chinense* Mill.	KH5521	S105	Gugijanamu	Fruit	Common cold	Boiling, brewing, decoction, infusion	Oral	41.46
						Eye disease	Boiling	Oral	12.20
						Gastroenteric disorder	Dried, tea	Oral	14.63
						Lack of energy	Infusion, tea	Oral	21.95
						Liver diseases	Infusion	Oral	9.76
	*Nicotiana tabacum* L.	KH5522	S110	Dambae	Leaf	Snakebite	Infusion	Topical	100.00
	*Physalis alkekengi* var. *francheti* (Mast.) Hort	KH5523	S127	Ggwari	Fruit	Tonsillitis	Infusion	Oral	100.00
	*Solanum melongena* L.	KH5524	S177	Gaji	Stem	Carpal tunnel syndrome	A sweet drink made from fermented rice, dried, infusion	Oral	50.00
						Tarsal tunnel syndrome	A sweet drink made from fermented rice, dried, infusion	Oral	50.00
	*Solanum nigrum* L.	KH5525	S178	Ggamajung	Aerial part	Abdominal pain	Infusion	Oral	4.55
						Anorexia	Infusion	Oral	4.55
						Indigestion	Infusion	Oral	31.82
					Fruit	Cough	Extraction	Oral	15.15
						Indigestion	Maceration	Oral	31.82
					Leaf	Indigestion	Maceration	Oral	31.82
						Urinary incontinence	Infusion	Oral	6.06
					Root	Indigestion	Maceration	Oral	31.82
					Stem	Pollakiuria	Infusion	Oral	6.06
						Urinary incontinence	Infusion	Oral	6.06
					Whole part	Cough	Infusion	Oral	15.15
						Excited delirium	Dried, infusion	Oral	10.61
						Herpes zoster	Infusion	Oral, topical	21.21
	*Solanum tuberosum* L.	KH5526	S179	Gamja	Tuber	Burn	Maceration, powder, rubbing	Topical	100.00
Staphyleaceae	*Staphylea bumalda* DC.	KH5527	S183	Gochunamu	Leaf	Facial nerve paralysis	Maceration	Topical	100.00
					Root	Facial nerve paralysis	Maceration	Topical	100.00
					Stem	Facial nerve paralysis	Maceration, raw	Topical	100.00
Sterculiaceae	*Firmiana simplex* (L.) W.F.Wight	KH5528	S75	Byeokodong	Leaf	Leukorrhea	Infusion	Oral	63.64
					Stem	Leukorrhea	Infusion	Oral	63.64
						Lumbago	A sweet drink made from fermented rice	Oral	18.18
						Pollakiuria	Infusion	Oral	18.18
Thelephoraceae	*Sarcodon aspratus* (Berk.) S. lto	KH5529	S166	Neungi	Whole part	Afterpain	Infusion	Oral	100.00
Tricholomataceae	*Armillariella mellea* (Vahl & Fr.) Karst.	KH5530	S20	Bbongnamubeoseot	Whole part	Glycosuria	Infusion	Oral	100.00
	*Tricholoma matsutake* (S. Ito. et Imai) Sing.	KH5531	S185	Songi	Whole part	Afterpain	Decoction, infusion	Oral	57.89
						Common cold	Decoction	Oral	21.05
						Tonsillitis	Infusion	Oral	21.05
Ulmaceae	*Ulmus davidiana* var. *japonica* (Rehder) Nakai	KH5532	S188	Neureupnamu	Bark	Boils	Dough, dried, maceration, powder	Topical	15.67
						Cancer	Infusion, maceration	Oral, topical	3.00
						Gastric ulcer	A sweet drink made from fermented rice, infusion, tea	Oral	2.07
						Gastritis	A sweet drink made from fermented rice	Oral	0.23
						Gastroenteric disorder	Dried, infusion, powder, tea	Oral	11.29
						Hemorrhaging	Powder	Topical	0.23
						Hemorrhoids	Infusion	Topical	2.76
						Indigestion	A sweet drink made from fermented rice, infusion	Oral	1.61
						Milk fever	Maceration	Oral, topical	3.92
						Pus	Dried, infusion, maceration, powder	Oral, topical	52.07
						Skin diseases	Maceration, powder	Topical	1.38
						Wound	Maceration, powder, raw	Topical	3.23
					Endodermis	Boils	Maceration	Topical	15.67
						Gastroenteric disorder	Infusion	Oral	11.29
						Pus	Maceration	Topical	52.07
					Rhizodermis	Boils	Dried, maceration, powder	Topical	15.67
						Gastroenteric disorder	Infusion	Oral	11.29
						Pus	Maceration	Topical	52.07
						Wound	Maceration, powder	Topical	3.23
					Root	Boils	Maceration	Topical	15.67
						Bruising	Maceration	Topical	0.46
						Cancer	Maceration	Topical	3.00
						Gastroenteric disorder	Dried, infusion	Oral	11.29
						Inflammation	Maceration	Topical	0.69
						Pus	Maceration	Topical	52.07
					Stem	Boils	Maceration	Topical	15.67
						Bone diseases	A sweet drink made from fermented rice, brewing	Oral	1.38
						Gastroenteric disorder	A sweet drink made from fermented rice, boiling, infusion	Oral	11.29
						Milk fever	Maceration	Topical	3.92
						Pus	Dried, maceration, powder	Topical	52.07
	*Ulmus parvifolia* Jacq.	KH5533	S189	Chamneureupnamu	Bark	Boils	Dough, maceration, powder, raw	Topical	100.00
Valerianaceae	*Patrinia villosa* (Thunb.) Juss.	KH5534	S119	Ttukgal	Leaf	Snakebite	Maceration	Topical	100.00
Violaceae	*Viola mandshurica* W.Becker	KH5535	S191	Jebikkot	Aerial part	Bruising	Maceration	Topical	16.13
						Hemorrhage	Rubbing	Topical	16.13
					Leaf	Snakebite	Maceration	Topical	16.13
					Whole part	Facial nerve paralysis	Maceration	Topical	22.58
						Sprain	Maceration	Topical	29.03
	*Viola verecunda* A.Gray	KH5536	S192	Kongjebikkot	Leaf	Dysentery	Seasoned cooked vegetables	Oral	100.00
Vitaceae	*Parthenocissus tricuspidata* (Siebold & Zucc.) Planch.	KH5537	S118	Damjaengideonggul	Stem	Chronic myofascial pain	Infusion	Oral	10.00
						Glycosuria	Infusion	Oral	40.00
						Lumbago	A sweet drink made from fermented rice	Oral	40.00
						Neuralgia	Decoction	Oral	10.00
	*Vitis coignetiae* Pulliat ex Planch.	KH5538	S194	Meoru	Fruit	Asthma	Extraction	Oral	33.33
						Bronchitis	Extraction	Oral	33.33
					Stem	Afterpain	Infusion	Oral	33.33
Zingiberaceae	*Zingiber officinale* Roscoe	KH5539	S199	Saenggang	Rhizoma	Arm pain	Decoction, dissolution, extraction	Oral	4.83
						Common cold	Boiling, decoction, dissolution, extraction, honey, infusion, tea	Oral	67.59
						Cough	Decoction, dissolution, extraction, infusion	Oral	22.76
						Leg pain	Decoction, dissolution, extraction	Oral	4.83

**Figure 2:**
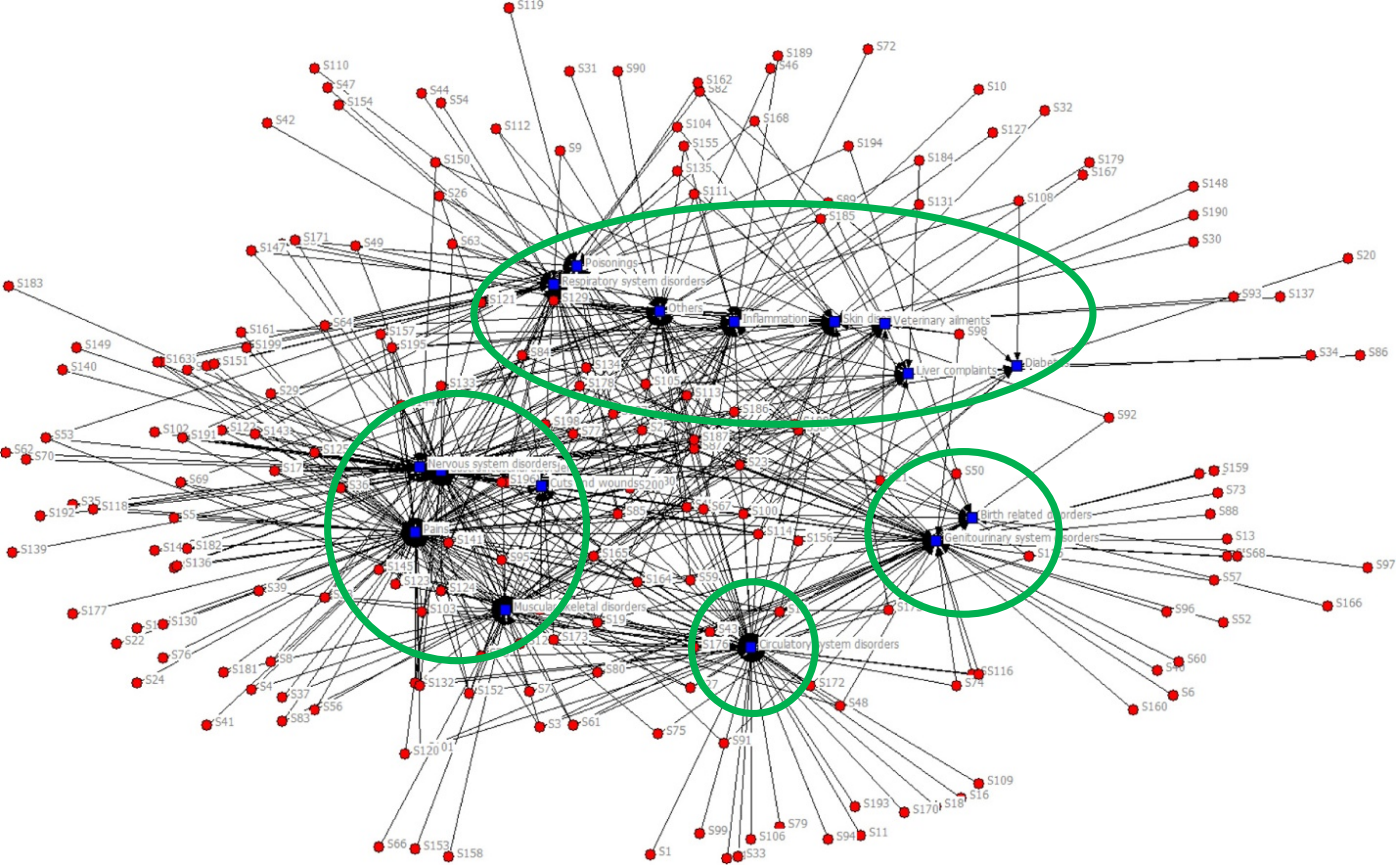
**The network relationship of ailments and plant species.**

*Abbreviation for Figure 2.

At present, these veterinary species do not affect the natural plant ecosystem of a national park anymore, as residents use a modern system of veterinary medicine to treat the diseases of domestic animals.

### Quantitative analysis

#### ICF

The ICF ranges from 0 to 1, where increasing values of this factor indicate a higher rate of informant consensus among the illness category.

The category with the highest degree of consensus from the informants was muscular-skeletal disorders (0.98). The ranking followed with remarks from informants related to concerns of pain (0.97), respiratory system disorders (0.97), liver complaints (0.97), and cuts and wounds (0.96). The lowest degree of consensus was birth-related disorders (Table 3). These results expose the fact that the residents living in a national park work in dry-field farming and laboriously gather products from the forests as they live in the mountainous region.

**Table 3:** **Category of ailments and their informant consensus factor (ICF) according to Heinrich et al.** [24]

Symptom and ailment categories	Taxons	Use citations	ICF
Muscular-skeletal disorders	49	2142	0.98
Pains	87	2942	0.97
Respiratory system disorders	46	1513	0.97
Liver complaints	15	447	0.97
Cuts and wounds	20	473	0.96
Inflammation	28	587	0.95
Genitourinary system disorders	52	1062	0.95
Circulatory system disorders	65	1250	0.95
Skin diseases and disorders	25	464	0.95
Gastrointestinal disorders	68	1257	0.95
Diabetes	10	165	0.95
Nervous system disorders	34	545	0.94
Veterinary ailments	22	300	0.93
Poisonings	26	321	0.92
Others	36	431	0.92
Birth-related disorders	14	138	0.91

Medicinal plants for treating disease with a higher degree of consensus can easily be harvested for treating medical ailments. As a result, the natural plant ecosystem of a national park may be partly destroyed in the near future. Therefore, through further study, proper steps to determine a wise alternative to protect them need to be considered.

#### FL

The FL is useful for identifying the key informants’ most preferred species in use for treating certain ailments. The FL values in this study varied from 1.0% to 100%. Generally, the FL of 100% for a specific plant indicates that all of the use-reports mentioned the same plant for specific treatment [33].

This study classified 57 species of plants with a FL of 100%, even without considering plants that were mentioned only once for better accuracy (Table 3). This information reveals that the informants had a tendency to rely on one specific plant species for treating one specific ailment than for several different ailments. These species possess a much higher potential for being gathered in Gayasan National Park.

Special attention for species conservation within the national park was given to important species (N, Np) with a FL above 100%, regarding the viewpoint of the number of times mentioned and the consensus level for the specific ailment, like *Solanum nigram* L. (68, 68) in being used for burns, *Fallopia japonica* (Houtt.) RonseDecr. for pollakiuria (60, 60), *Colocassia esculenta* (L.) Schott (15, 15) for sterility, *Euonymus sachalinensis* (F. Schmidt) Maxim. (13, 13) for hemorrhoids, and *Staphylea bumalda* DC. (13, 13) for facial nerve paralysis (Table 2).

### INA between categories of ailments and medicinal plants

INA has originally analyzed social phenomenon and its trends through the network of components [34]. Our research has attempted to analyze the interrelationship between aliments and medicinal plants recorded within these communities.

In relation to the network of ailments and medicinal plants, the position of the ailments is distributed into four main groups, where each circle represents four different groups of ailments (Figure 2).

Accordingly, the first group is positioned in the upper section of Figure 2 and consists of poisonings, respiratory system disorders, others, inflammation and so on.

The second group is located in the bottom left section and consists of nervous system disorders, gastrointestinal disorders, cuts and wounds, and pains. This group of disorders will require a deeper analysis in order to determine the overlapping use of medicinal species for these ailments.

The third group is positioned on the left side and consists of birth-related disorders and genitourinary system disorders. These results depict that the same medicinal plants used for treating birth related disorders and genitourinary system disorders have a close relationship.

Finally, the circulatory system disorders are the fourth group and are positioned in the bottom section.

## Conclusion

These days, the natural conservation of a national park has focused strongly to preserve bio-resources, which are regarded as vital to its environment. However, the natural conservation of a national park is practically determined by the interrelationship between the bio-resources and its utilization by residents.

In relation to this point, this study contains a major focus to obtain the basic data regarding the natural conservation of a plant ecosystem, through the analysis and investigation of traditional knowledge for medicinal plants being used by residents of Gayasan National Park.

Conclusions regarding the preservation of a sustainable natural plant ecosystem within a national park obtained the following results:

First, the high percentage of medicinal plants among flora means that the total amount of medicinal plants gathered by residents may negatively affect the conservation of a natural plant ecosystem within a national park. In order to conserve the plant ecosystem, residents need an adaptable health care system for treating their ailments, instead of relying solely on ethnomedicinal therapies.

Second, residents mentioned quite often that they used medicinal plants to treat their own medical conditions. Therefore, it is necessary to prepare a plan of action for conserving the popular medicinal plants.

Third, medicinal plants for treating the category of disease with a higher degree of consensus can be actively harvested for treating medical problems. The over collecting of these medicinal plants will disrupt the plant ecosystem of Gayasan National Park in the near future.

Fourth, 57 species of plants with a FL of 100% possess a much higher potential for being gathered in the region and it is vital to protect the overuse of these medicinal plants.

Fifth, the results of INA will provide an appropriate plan for the sustainable preservation of a national park through continued study.

Therefore, these particular species need to be conserved for a balanced plant ecosystem within the park.

Consequently, through further study using these results, proper steps need to be established for preparing a wise alternative to create a sustainable natural plant ecosystem for Gayasan National Park and other national parks.
